# Dutch, UK and US professionals’ perceptions of screening for Barrett’s esophagus and esophageal adenocarcinoma: a concept mapping study

**DOI:** 10.1186/s12885-023-11583-x

**Published:** 2023-11-14

**Authors:** Jasmijn Sijben, Linda Rainey, Yonne Peters, Rebecca C. Fitzgerald, Sachin Wani, Jennifer M. Kolb, Mireille J. M. Broeders, Peter D. Siersema

**Affiliations:** 1 Department of Gastroenterology and Hepatology (Route 455), Radboud University Medical Center, Geert Grooteplein-Zuid 8, 6500 HB Nijmegen, the Netherlands; 2 Department for Health Evidence, Radboud University Medical Center, Nijmegen, The Netherlands; 3 Early Cancer Institute, University of Cambridge, Cambridge, UK; 4 Division of Gastroenterology and Hepatology, University of Colorado Anschutz Medical Center, Aurora, USA; 5 Division of Gastroenterology, Hepatology and Parenteral Nutrition, VA Greater Los Angeles Healthcare System, David Geffen School of Medicine at UCLA, Los Angeles, USA; 6 Dutch Expert Centre for Screening, Nijmegen, The Netherlands

**Keywords:** Barrett’s esophagus, Esophageal adenocarcinoma, Early cancer diagnosis, Screening, Physician’s practice patterns

## Abstract

**Background:**

Novel, less-invasive technologies to screen for Barrett’s esophagus (BE) may enable a paradigm shift in early detection strategies for esophageal adenocarcinoma (EAC). Understanding professionals’ perspectives on screening is important to determine how to proceed. We aimed to explore and compare professionals’ perceptions of screening for BE and EAC screening in three countries.

**Methods:**

In this study, 29 Dutch, 20 British and 18 American health care professionals (clinicians, researchers and policy makers) participated in concept mapping: a mixed-methods consensus building methodology. Statements on perceived barriers, facilitators, advantages, disadvantages, implications or worries associated with screening for BE and EAC were collected in asynchronous digital brainstorm sessions. Subsequently, participants sorted the statements into groups according to thematic similarity and assessed the relevance of each statement in evaluating the acceptability of BE and EAC screening. Multidimensional scaling and cluster analysis were used to map the associations between generated statements.

**Results:**

Professionals across three countries identified eight consistent themes that relate to their perceptions of screening for BE and EAC: (1) Benefits, (2) Harms, (3) Clinical effectiveness concerns, (4) Screening population, (5) Screening modality, (6) Resources, (7) Ownership, and (8) Public communication. Dutch and American professionals prioritized the potential health benefits of screening but also questioned clinical impact. In contrast, British participants prioritized identification of the screening population and suitable test.

**Conclusions:**

Most professionals see potential in less-invasive screening tests for BE and EAC but underline the need to define the target screening population and determine benefits and harms before widely employing them. Successful implementation will require thoughtful consideration of the involvement of general practitioners, readiness of endoscopy and pathology services, balanced public communication, and country-specific regulations.

**Supplementary Information:**

The online version contains supplementary material available at 10.1186/s12885-023-11583-x.

## Background

Dutch, British, and American populations are among the most affected by esophageal adenocarcinoma (EAC), with respectively 2600, 8600, and 15,000 individuals predicted to be diagnosed annually in the next decade [1]. Endoscopic treatment of dysplasia and intramucosal cancer in Barrett’s esophagus (BE), the precursor of EAC [2, 3], prevents progression to advanced cancer and reduces mortality [4, 5]. European and American gastroenterology guidelines therefore recommend to screen at-risk individuals for the presence of BE, followed by regular surveillance endoscopies to detect and treat early neoplasia [6–8]. The at-risk population is defined as individuals with gastroesophageal reflux disease [GERD] combined with other risk factors such as age > 50 years, male sex, Caucasian race, obesity, and family history, although the precise definition of risk factors varies among guidelines. Screening currently depends on individual clinicians’ screening decisions with societal guidelines as guidance: no public organizations, such as the US Preventive Services Task Force or the UK National Screening Committee, recommend screening for BE or EAC screening at the moment.

Research on less invasive tests that may enable widespread screening for BE or EAC is expanding. The Barrett’s oESophagus Trial 3 (BEST3) showed that, in individuals with GERD, the offer of Cytosponge-TFF3 testing results in improved detection of BE compared with usual care (rate ratio of 10.6) [9]. Other alternatives, such as transnasal endoscopy or breath analysis, have been studied in regional randomized controlled trials (RCTs) or case–control studies in the US and the Netherlands [10, 11]. As screening for EAC currently depends on health care professionals’ decisions, their endorsement is crucial to facilitate successful development and implementation of screening methods.

In previous surveys, 70% of health care providers believed that BE screening with upper endoscopy is effective for early EAC detection, but only 38–56% believed it to be cost-effective, and few (22%) believed it would reduce all-cause mortality [12, 13]. In another American survey, 85% of gastroenterologists believed BE screening to be less efficacious than colorectal cancer screening [14]. General practitioners (GPs) reported additional concerns, such as difficulty in identifying which patients to screen, unavailability of open access endoscopy (GP-initiated endoscopy without gastroenterologist consultation), time limitations, financial cost, lack of evidence, and poor patient acceptance [12, 15, 16].

Insight into health care professionals’ perceptions of novel, and less invasive, screening tests is scarce since these technologies are relatively new. Furthermore, previous studies only investigated acceptance of screening among GPs and gastroenterologists, missing the opportunity to collect input from professionals potentially involved in diagnostic services (e.g., pathologists), treatment of screen detected EAC (e.g., surgeons, oncologists, radiotherapists), researchers (e.g. epidemiologists and ethicists), and public health officers. Involving experts from different fields is needed to comprehensively assess potential issues associated with the acceptability of novel screening tests.

The aim of this study was to explore clinicians’, researchers’, and policy makers’ perceptions of screening for BE or EAC (including currently recommended and novel screening methods). Moreover, we aimed to explore whether there were differences across countries (Netherlands, UK, US) and different professions.

## Methods

### Study design

Health care professionals participated in digital concept mapping. This is a standardized mixed-methods approach that integrates qualitative procedures to generate a wide array of ideas and quantitative methods to organize these ideas and expose interconnections and core areas [17]. In contrast to focus groups or surveys, the concept mapping process is participant-driven and involves stakeholders directly in the interpretation of the results. Ethics approval was acquired from the regional ethics committee CMO Arnhem-Nijmegen in the Netherlands (Ref no. 2021–7354). All participants provided informed consent before the start of the study.

### Sampling strategy

 We purposefully selected professionals that were involved in esophageal cancer care or specialized in cancer screening, including clinicians, researchers, and policy makers/advisors. We aimed for diversity in practice setting (e.g., public health, primary care, or hospital), and geographical region. We invited professionals who were part of the professional network of members of the research team (RF, SW, JK, MB, PS). We sent personal invitations to Dutch and American professionals and used pre-existing infrastructures (i.e., the BEST3 and Oesophageal Cancer Clinical and Molecular Stratification consortia) to send a general invitation to relevant British professionals. For representation of policy makers’ perspectives, we invited professionals working on BE or EAC screening guidelines (US/UK) and professionals involved in established cancer screening programs (NL). Invitations were sent by e-mail until at least 15 professionals per country agreed to participate, since a sample size of 10 to 40 participants is recommended for this method [17].

### Concept mapping procedures

**Fig. 1:**
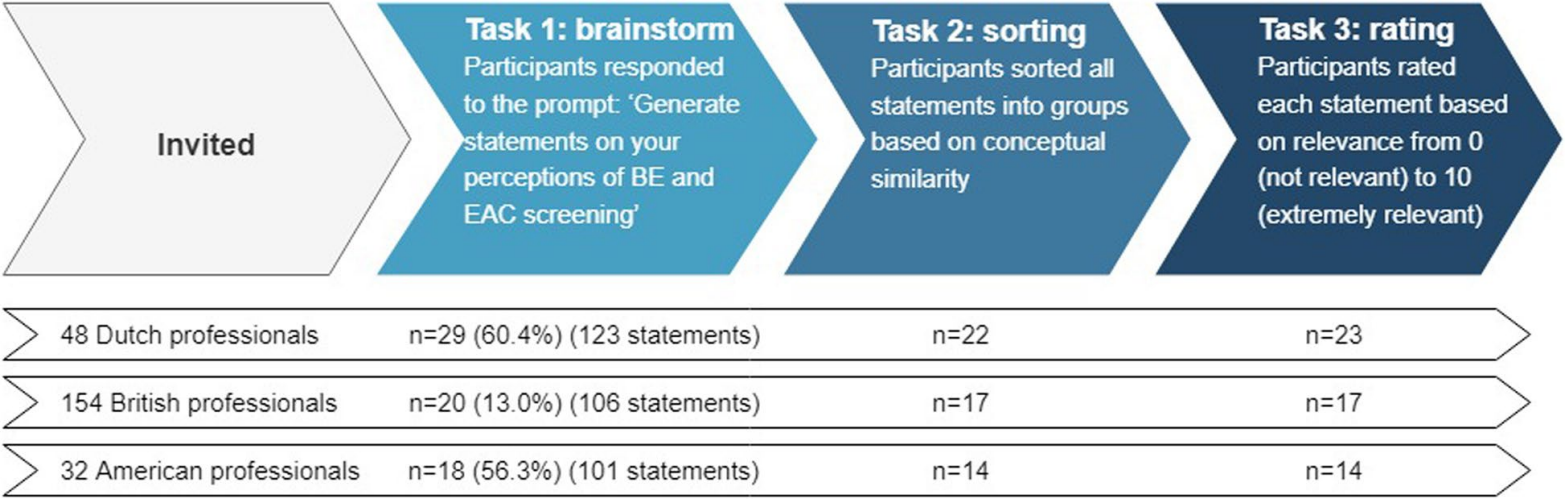
Flow chart illustrating data collection steps and participants

Data were collected digitally from March 2021 to October 2021 in three phases: brainstorming, sorting, and rating (Fig. 1). For brainstorming, participants were presented with the following ‘focus prompt’ by e-mail: *‘Generate statements on your perceptions (e.g., perceived barriers, facilitators, advantages, disadvantages, implications or worries) of Barrett’s esophagus and esophageal adenocarcinoma screening’*. Participants were instructed to individually brainstorm for ten minutes and to write down concise statements that contained a single idea or thought and submit them to the researcher by e-mail. Spelling or grammatical errors were corrected, and duplicate statements were removed (by JS). A second researcher (YP, MB, or PS) was consulted in case of uncertainty. The unique verbatim statements from each country were subsequently uploaded onto three separate web-based platforms for the Netherlands, UK and US using Concept Systems software version 4.0175, Concept Systems, Inc. (Ithaca, NY). All respondents were provided with a URL that directed them to the platform of their country, e.g. each British participant was presented with the statements generated by all other British participants. Participants were first instructed to sort the statements into groups based on perceived thematic similarity, and to provide a label for each group (see Additional file 1 for the specific wording of the instructions). If a participant sorted statements based on value (e.g., ‘important’ vs ‘not important’) or failed to sort at least 95% of the statements, their data were removed from the analysis (to facilitate the identification of meaningful relationships among statements and ensure comprehensive task engagement) [17]. In the second task of the same survey, participants rated all statements on an eleven-point Likert-type scale using the following question: ‘*When evaluating the acceptability of Barrett's esophagus and esophageal adenocarcinoma screening, how relevant is this statement or question?*’ (ranging from 0 = not at all relevant to 10 = very relevant). Data from participants that rated at least five statements were included, based on the requirement settings of the concept mapping software.

### Data analysis

We applied multidimensional scaling and hierarchical cluster analysis to create cluster-rating maps using the Concept Systems software (see Additional file 1 for a detailed description). The cluster-rating maps provide a visual representation of the participants' sorting data with each point representing a statement and the distance between each point reflecting how frequently the statements were sorted together by participants. The map also depicts mean rating values, relative to the other clusters, as stacked layers [17]. We applied pattern match analysis to compare the mean relevance ratings of each cluster between two subgroups (i.e., ‘clinicians’ vs ‘researchers and policy makers’). Pearson *r* coefficient (ranging from 0 to 1) was used to describe correlations between the mean ratings. A low correlation indicates differences in priority-setting between subgroups.

### Integration and interpretation

Interpretation of the final cluster-rating maps was conducted in digital meetings with the representative team members from each country (by JS, LR, YP, RF, SW, JK, MB, PS). To provide an integrated overview of Dutch, British, and American participants’ perspectives and to explore similarities and differences, one researcher (JS) colour coded similar clusters across the three countries. The maps were subsequently discussed (by JS, LR, YP, MB, PS) to come to a final categorization of each cluster in overarching themes which are outlined in Table 1.

**Table 1:** Themes related to screening for esophageal adenocarcinoma and identified categories per country

Overarching theme	Integrated content summary	Category	Country	No. of layers^a^	Exemplary statements
Potential benefits	*Screening may lead to prevention or early detection of EAC, fewer invasive esophagectomies, increased opportunity for endoscopic resection or ablation. This may lead to decreased EAC-related morbidity and mortality and improved quality of life*	Potential health benefits	NL	*****	“Early detection leads to a shift in the type of treatment (e.g., more EMRs/ESDs, fewer esophagectomies) and therefore fewer major complications” (pathologist)
		Impact of screening?	US	*****	“Although EAC is a low incidence cancer in the US, its high morbidity may justify screening” (gastroenterologist) “Most patients with BE will never develop EAC” (surgeon)
		Clinical Need	UK	**	“The attractive theoretical benefit of detecting more cases of BE and applying endoscopic therapy to reduce EAC incidence” (gastroenterologist)
Harms	*Endoscopic complications such as perforation and bleeding, overdiagnosis resulting in too aggressive treatment, and psychological harm such as cancer worry*	Harms of screening	NL	**	“The risk of overtreatment which results in more complications” (researcher)
		Patient fear	US	*	“Patients hear worst-case stories from friends and relatives about endoscopic procedures” (gastroenterologist) “How will we reassure patients diagnosed with very low risk BE?” (policy maker)
		Impact on individual	UK	*	“Barrett’s screening will provoke unnecessary esophagectomies of patients overdiagnosed with non-lethal early esophageal cancers” (pathologist) “Increasing anxiety in screening participants awaiting test results” (GP)
Clinical effectiveness	*Obstacles that might diminish the impact of screening i.e., high miss rate in patients without reflux complaints, relatively low population incidence of EAC, knowledge gaps in natural history of BE, and misdiagnosis of BE due to inadequate endoscopic assessment*	Legitimacy	NL	****	“Screening always leads to risk and harm, sometimes to some good and rarely to more good than risk and harm” (ethicist) “Adding an easily accessible screening test to an unfavourable benefit-risk ratio is like a fish trap with bait: you can enter, but never leave” (policy advisor)
		BE surveillance issues	US	***	“A system akin to colorectal ‘adenoma detection rate’ could be developed for detection of BE and associated lesions” (pathologist) “Will it lead to widespread misdiagnosis of BE (i.e., normal Z-line epidemic?)” (gastroenterologist)
		Natural history unknowns	UK	**	“Don’t we still risk missing the more aggressive cancers that occur rapidly and are these the ones most associated with poor outcomes?” (researcher)
		Clinical effectiveness concerns	UK	*	“Lack of documented mortality benefit BE surveillance will translate to a lack of documented benefit BE screening” (pathologist)
Screening modality	*The screening modality needs to be minimally invasive and should have high specificity, sensitivity and public acceptance*	Design screening method	NL	****	“Screening should be as non-invasive as possible, transnasal endoscopy is too invasive for individuals without symptoms in my opinion” (gastroenterologist)
		Screening modality	UK	***	“A home kit test might become more popular among the general population rather than a test in hospital” (GP)
Screening population	*Elaboration on who should potentially be invited for screening and how to select these individuals*	Screening population	UK	*****	“Offering screening to participants with higher pre-test probability than the general population would be a facilitator” (epidemiologist)
		Target population identification	NL	*	“Selecting the population at risk is complicated and requires training for doctors or other health care personnel” (GP) “The inclusion criterion ‘white male’ is a form of ethnic discrimination, which is politically questionable” (gastroenterologist)
Ownership	*Views on which organization should take ownership of patient identification and testing, endoscopy and pathology services*	Operationalization and partnership	US	*****	“How do we empower GPs to identify patients for screening?” (gastroenterologist)
		Recommended service organization	UK	***	“Read-out of any test should be ubiquitously feasible, not only in a few centralized labs” (gastroenterologist)“A multidisciplinary team is required to support the screening service and discuss it with patients” (surgeon)
Resources	*Worry about the increase in workload for primary care, NHS services (UK), endoscopy units and pathologists with the introduction of BE or EAC screening. Perceived methods for increasing cost-effectiveness and acquiring funding*	Cost-effectiveness	NL	*****	“Screening with a non-invasive test aimed at detecting BE with (high-grade) dysplasia may be cost-effective” (researcher)
		Resources and reimbursement	US	****	“Convincing insurance companies that screening for BE once in a lifetime should be a covered benefit paid in full” (gastroenterologist)
		Capacity	NL	***	“The current endoscopy capacity is insufficient for the expected increase in BE surveillance” (gastroenterologist)
		Roll-out concerns	UK	**	“GP surgeries and most NHS services already under pressure to deliver services” (researcher) “Concern that screening would not reach the hardest-to-reach population groups at most risk” (epidemiologist)
Public communication	*Ideas for raising awareness of esophageal cancer among the public and warnings about inappropriate framing of BE*	Public awareness	US	***	“Heartburn is a common symptom and people can self-medicate for months to years without seeking help” (oncologist) “Improving public awareness by appropriate labelling on bottles of over-the-counter PPIs and in advertising by makers of these compounds” (pathologist)
		Patient education	UK	**	“There is need for patients to understand the implication of a positive screening test…. What would follow?” (GP)

*EMR* endoscopic mucosal resection, *ESD* endoscopic submucosal dissection, *BE* Barrett’s esophagus, *EAC* esophageal adenocarcinoma, *GP* general practitioner, *NHS* National Health Service, *NL* Netherlands, *UK* United Kingdom, *US* United States

^a^Reference to the number of stacked layers on the concept map, where more layers indicate higher mean rating values for that cluster

## Results

### Participants

A total of 67 health care professionals participated in the brainstorm activity. Figure 1 shows participant retention rates across the different concept mapping steps (see Additional file 2 for information on non-responders). Sorting data from seven participants were excluded for analysis due to failure to sort at least 95% of the statements (*n* = 3), or not sorting based on thematic similarity (*n* = 4). Table 2 shows participants’ characteristics across the three countries, with the majority being male (64.0%) working in various health care settings with over 10 years of experience in their field.

**Table 2:** Participant characteristics

	**Netherlands**	**United Kingdom**	**United States**
Gender (% female)	55.2	25.0	27.8
Age (median, range)	48 (33–68)	44.5 (33–56)	50.5 (34–68)
Profession type			
*Clinicians (n, %)*	16 (55.1)	14 (70.0)	12 (66.7)
General practitioner (n)	2	3	1
Physician extender (n)	2	0	1
Gastroenterologist (n)	8	4	6
Oncologist (n)	1	2	3
Surgeon (n)	2	5	1
Radiotherapist (n)	1	0	0
*Service specialties (n, %)*	3 (10.3)	4 (20.0)	3 (16.7)
Pathologist (n)	3	2	3
Radiologist (n)	0	2	0
*Researchers (n, %)*	3 (10.3)	2 (10.0)	1 (5.5)
*Policy advisors (n, %)*	7 (24.3)	0 (0)	2 (11.1)
Years of experience (median, range)	10 (3–33)	15 (3–29)	16 (3–37)

### Statement generation

In response to the focus prompt, Dutch professionals generated a total of 123 unique statements, British professionals 106, and American professionals 101. The final list of statements and clusters is available in Additional file 3, stratified by country and sorted by mean cluster/statement relevance rating. 

### Cluster-rating maps

#### The Netherlands

**Fig. 2:**
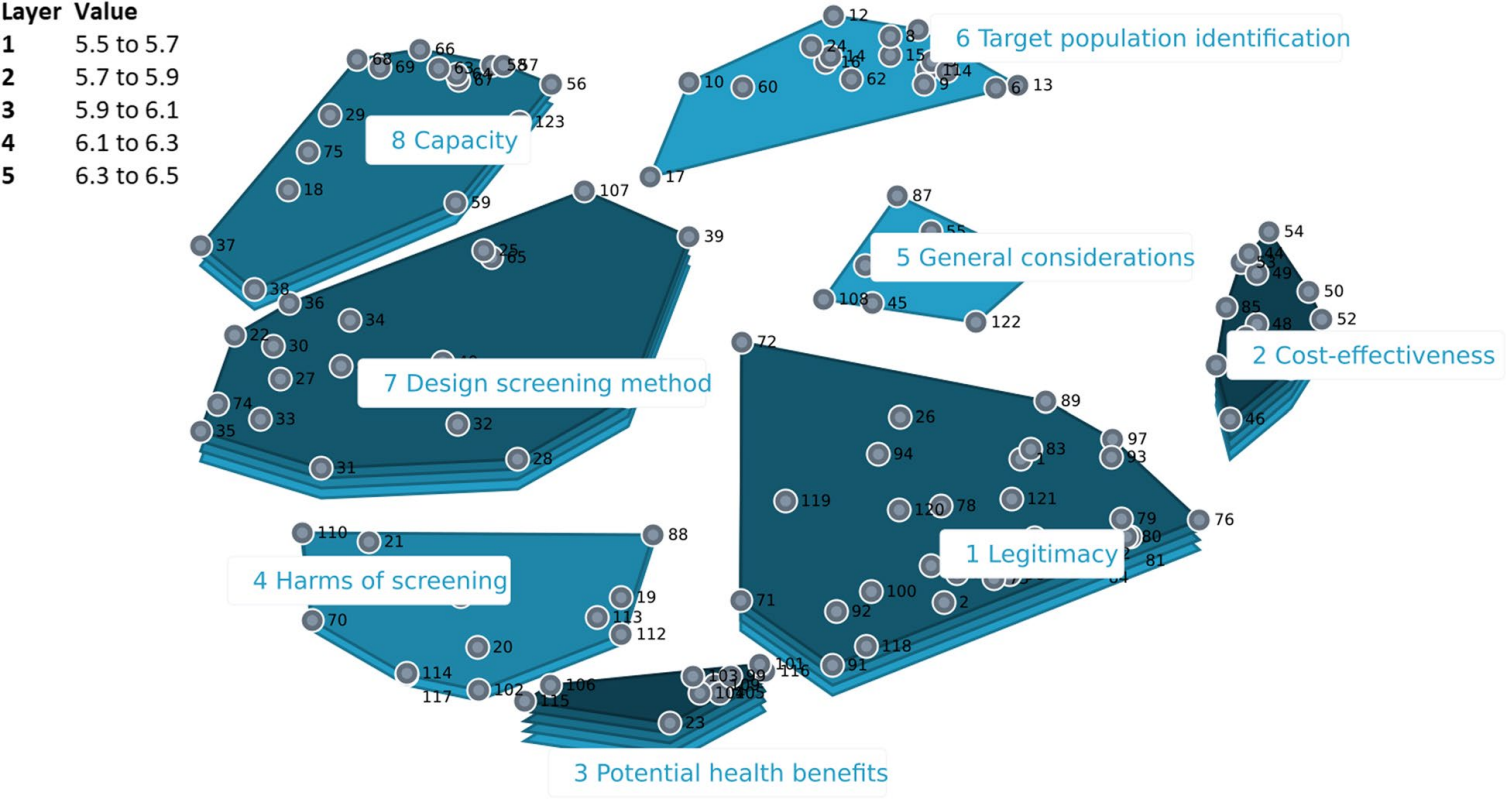
Cluster rating map the Netherlands. The cluster rating map provides a visual representation of the participants’ sorting data with each point representing a statement and the distance between each point reflecting how frequently the statements were sorted together by participants. Statements that were sorted together more frequently are positioned closer to each other on the map. The map also depicts mean rating values, relative to the other clusters, as stacked layers where more layers represent higher mean rating values for that cluster

The cluster-rating map which best fit the Netherlands data consisted of eight categories (Fig. 2). ‘*Potential health benefits’* was the category with the highest mean relevance rating (6.51 out of 10) and the category ‘*Target population identification’* had the lowest relevance rating (5.46). The ‘*Legitimacy*’ cluster (mean rating of 6.15) contained statements about ethical issues and the risk/benefit balance that were unique for the Dutch brainstorm input (exemplary statements for each category are shown in Table 1).

#### United Kingdom

**Fig. 3:**
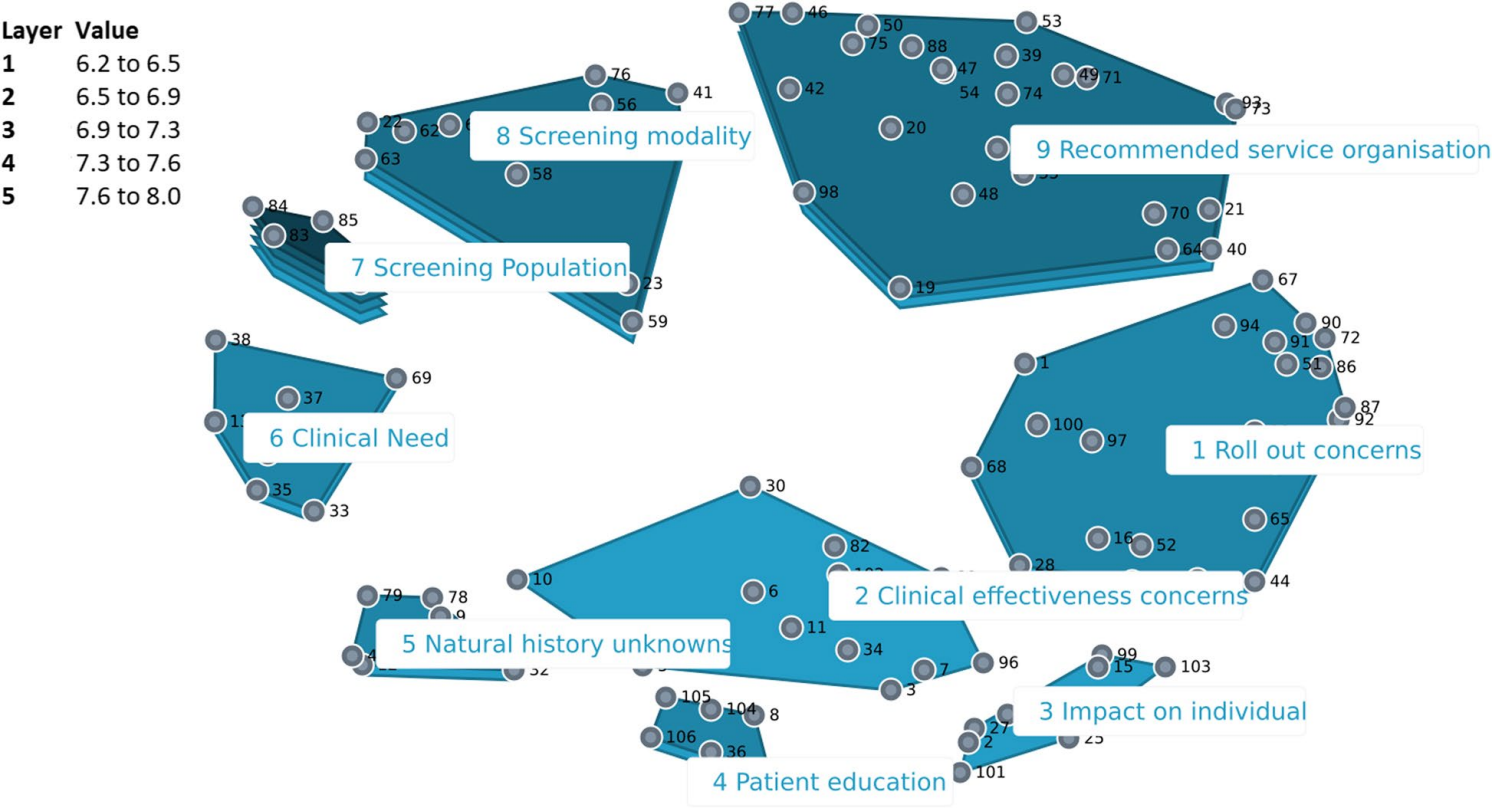
Cluster rating map United Kingdom

The cluster-rating map providing the best fit for the UK data consisted of nine categories (Fig. 3). The category *‘Screening population’* was considered most important with a mean relevance rating of 8.01. British participants regarded the category *‘Impact on individual’* as least important for acceptability of BE and EAC screening (mean rating of 6.16). This concept map contained a notably large number of statements in the categories *‘Recommended service organization’* and *‘Roll-out concerns’*.

#### United States

**Fig. 4:**
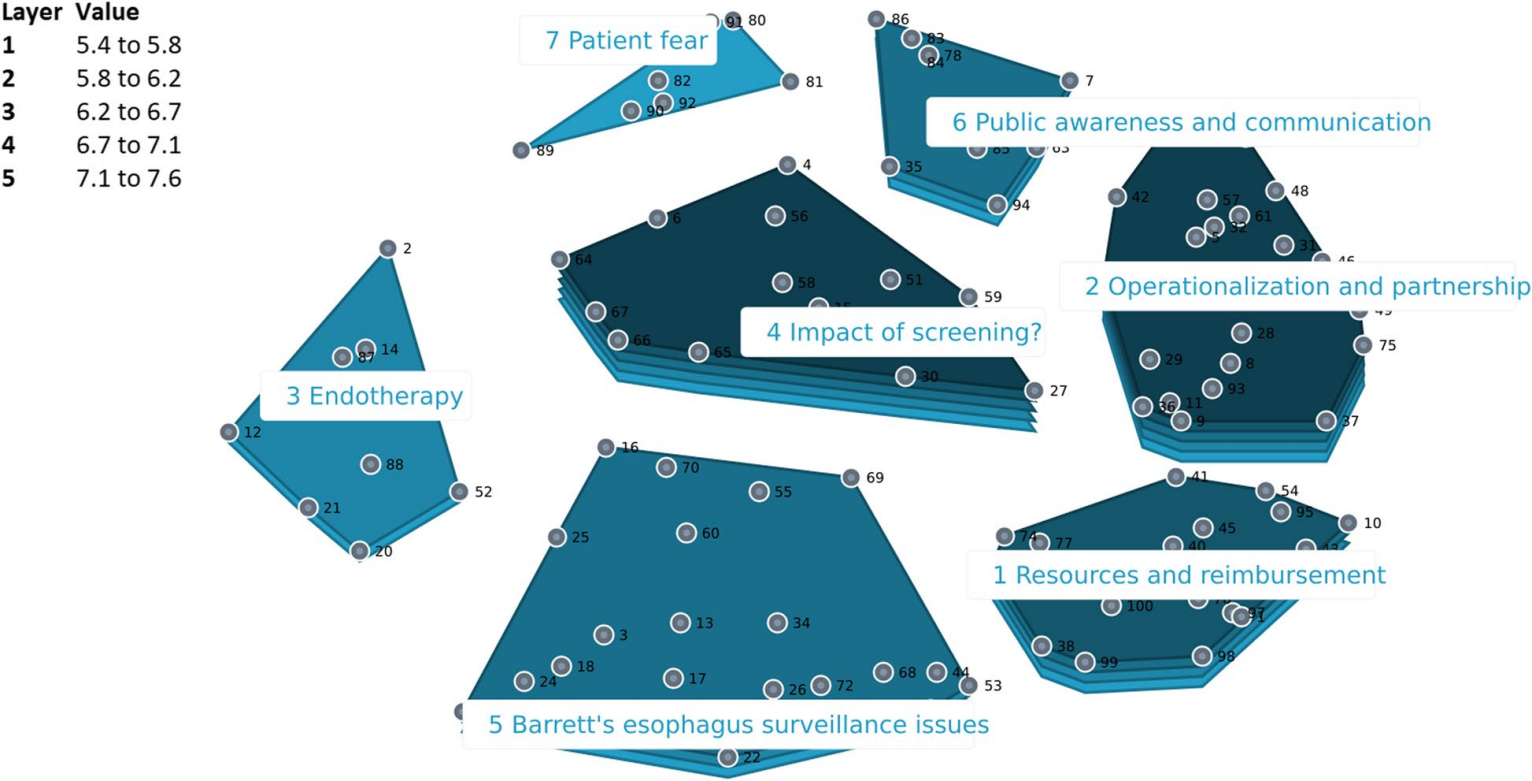
Cluster rating map United States

The cluster-rating map which best fit the US data consisted of seven categories (Fig. 4). The category with the highest mean priority rating was *‘Impact of screening?’* (7.57 out of 10) and *‘Patient fear’* was considered least relevant (5.35 out of 10). The category *‘Barrett’s esophagus surveillance issues’* was unique for the US concept map and included statements about endoscopic and pathological interobserver variability and natural history knowledge gaps.

### Integration

Eight core themes were identified after comparing and integrating the findings on the Dutch, British and American cluster rating maps: (1) Benefits, (2) Harms, (3) Clinical effectiveness concerns, (4) Screening population, (5) Screening modality, (6) Resources, (7) Ownership, and (8) Public communication (Table 1).

### Cultural variation among countries

American participants did not identify categories on the screening population and modality, while Dutch participants did not identify public awareness and ownership as themes relevant for BE and EAC screening. Dissimilarities were also present in prioritizing, e.g., British professionals prioritized the category *‘Screening population’*, whereas this topic had the lowest priority rating among Dutch professionals. Variation in screening organization and insurance infrastructure resulted in country-specific statements such as: “Given the freedom for individuals to choose a health insurer, including switching to another insurer, screening will need to be funded by the Ministry of Health, Welfare and Sports” (NL), “Screening within the National Health Service should be organized by national roll-out” (UK), and “Upper endoscopy costs money to most patients of non-Medicare age” (US).

### Professional background variation

**Fig. 5:**
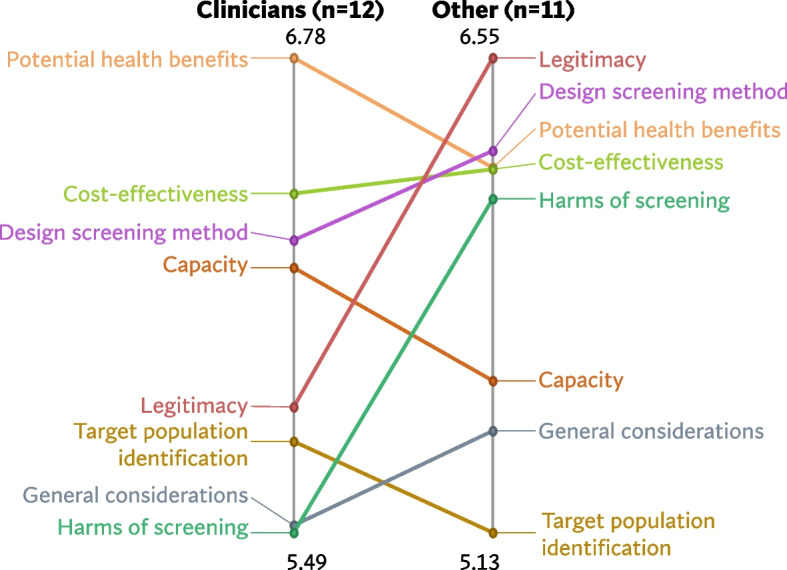
Pattern match graph showing rating patterns of Dutch clinicians and other professionals (e.g., researchers and policy makers)

**Fig. 6:**
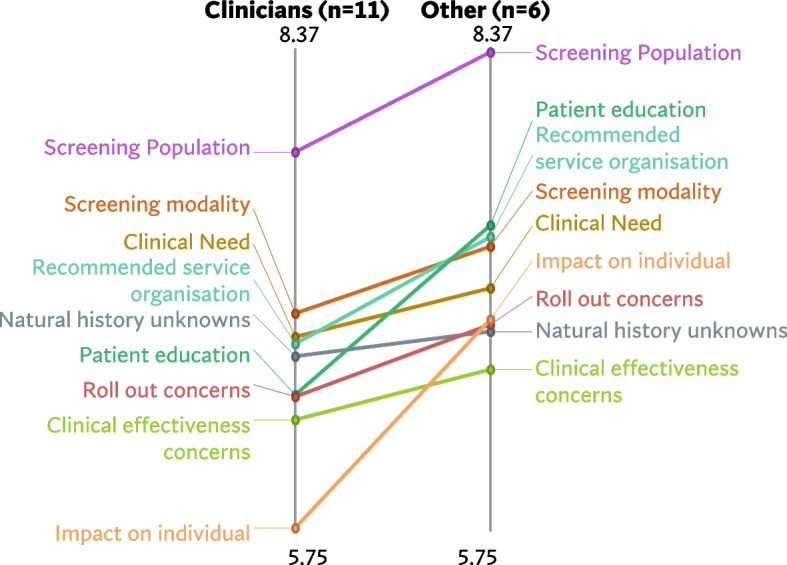
Pattern match graph United Kingdom

**Fig. 7:**
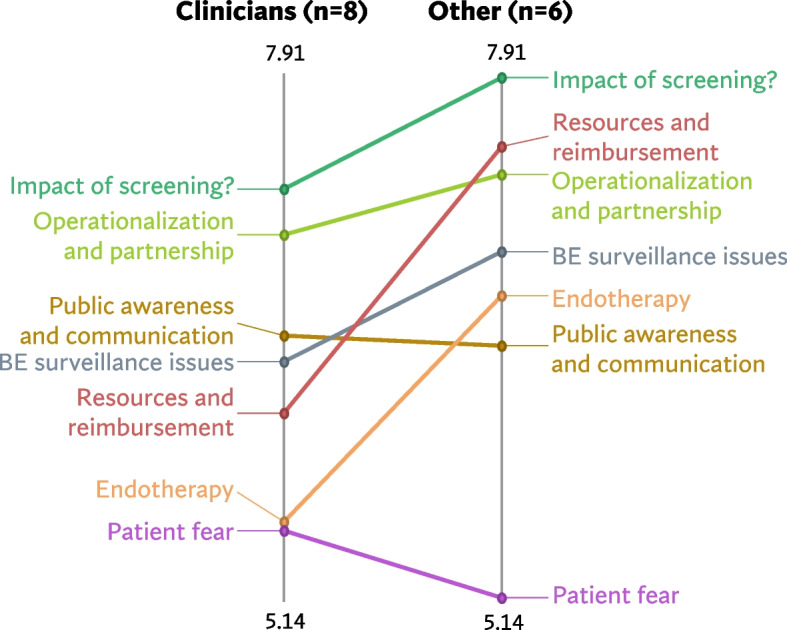
Pattern match graph United States

Figures 5, 6 and 7 shows a comparison of the rating patterns of clinicians versus other professionals. Dutch clinicians, in evaluating the relevance of statements for the acceptability of BE and EAC screening, assigned relatively higher scores to statements within the categories *‘Potential health benefits’*, *‘Capacity’* and *‘Target population identification’*. In contrast, Dutch researchers and policy makers assigned higher relevance scores to statements within the themes *‘Legitimacy’* and *‘Harms of screening’* (with mean cluster ratings ranging from 5.46 to 6.51). Clinicians and other professionals had similar rating patterns in the UK and US data.

## Discussion

This study provides a unique overview of Dutch, British, and American professionals’ perspectives on screening for BE and EAC. Involving diverse stakeholders has contributed to a wide range of perspectives on screening emerging from the brainstorm sessions (Table 1). Variation in submitted statements between professionals from the Netherlands, UK, and US shows that new insights can be gained from international exchange of views and ideas for future screening strategies.

### Comparison with the literature

Professionals emphasized in their statements within the categories *‘Benefits’* and *‘Clinical effectiveness’* that the potential of screening to prevent EAC and reduce EAC-related mortality is currently theoretical and lacks empirical evidence. This finding is consistent with a recent American survey involving 315 gastroenterologists and primary care providers, where the majority of participants expressed the need for more data evaluating both the benefits (69% concurred) and potential harms (62% concurred) of BE screening, including insights from RCTs (84% concurred) [12]. The deficiency in supporting evidence likely contributes to professionals’ low belief in the effectiveness of BE and EAC screening, as reported in earlier surveys [12–14]. These perspectives are understandable, given that the impact of screening on EAC-related mortality remains unstudied [6]. Robust evidence will be required in order for healthcare professionals to accept BE or EAC screening. To the best of our knowledge, there is one ongoing RCT, BEST4, aiming to assess whether offering the Cytosponge-TFF3 to patients on medication for heartburn symptoms could lead to an improvement in EAC-associated mortality [18]. However, results are not expected until 2035.

Participants’ statements regarding the prerequisites for an acceptable screening modality were largely congruent with our prior analysis of expert opinion articles, namely: minimally-invasive and acceptable to the public, high sensitivity and specificity, easily administered, and low costs [19]. Evaluation of the statements on an item-level reveals that most professionals perceived the Cytosponge-TFF3 and breath test as more acceptable modalities for large-scale screening compared to conventional endoscopic screening. This sentiment broadly aligns with the general public’s perspective, though some individuals express reservations about the anticipated discomfort associated with the Cytosponge-TFF3 [20, 21]. Notably, none of the professionals suggested the use of transnasal endoscopy as a screening modality, reflecting its limited clinical adoption since being endorsed as an alternative for conventional endoscopy by the American College of Gastroenterology in 2016 [6, 22]. The public also appears to perceive transnasal endoscopy as the least preferable test modality [23].

Our study findings resonate with previous surveys showing that GPs perceive time constraints and difficulty identifying suitable candidates for BE and EAC screening as barriers [12, 13, 16]. A prior American survey reported that 58% of GPs encountered challenges in determining who should undergo upper endoscopy for BE screening, compared to only 16% of gastroenterologists [12]. One potential explanation is that the published literature on risk factors and screening for EAC has predominantly been confined to gastroenterology journals. Furthermore, the underrepresentation of GPs in our study (see Additional file 2) might suggest a lower inclination towards conducting BE or EAC screening, or perhaps a perception that it falls outside their designated scope of practice. In this light, gaining a deeper understanding of GPs’ perspectives on their role in shared decision-making regarding BE and EAC screening, as well as their involvement in administrating screening tests, is crucial. This is especially true in the context of implementing a primary care-based tool like the Cytosponge-TFF3. We therefore recommend conducting an additional qualitative study focused on GPs’ role preferences, information needs and facility requirements.

### Differences across countries

The divergence in terminology between American participants, who referred to the public communication category as ‘public awareness,’ and British participants, who labeled it as ‘patient education,’ likely reflects country-specific approaches to initiating cancer screening. In Europe, screening policies typically adhere to a publicly coordinated and systematic approach, incorporating proactive screening invitations and reminders that necessitate balanced educational materials [24]. In the US, screening practices adopt a more decentralized model relying on individuals taking the initiative. Individual healthcare providers play a pivotal role in determining the most appropriate screening tests and engaging in shared decision-making. This distinction may explain why American professionals suggested strategies such as using social media platforms and incorporating labels on over-the-counter acid-suppressants to enhance public awareness of BE and EAC.

Part of the British participants were connected to the previously described BEST3 trial [9]. This real-world experience provided them with valuable insights into practical barriers and facilitators of BE screening. These encompassed securing funding, ensuring easy and equitable community access, developing high-quality public communication materials, training personnel in the Cytosponge-TFF3 procedure, and ensuring adequate pathology and endoscopy services for the follow-up of screening participants. Consequently, fostering collaboration with the UK to draw from their experiences and potentially launching a multinational screening trial could prove valuable for the Netherlands. This is because the Netherlands has a similar approach to cancer screening but limited experience with large-scale BE or EAC screening trials in primary care settings [10, 11].

### Strengths and limitations

A major strength of our study using a systematic combination of qualitative and quantitative methods is that we engaged participants in the analytic stages of the study. This minimized individual interpretation bias. Additionally, the purposeful selection of professionals allowed us to collect input across different organizational levels and profession types in a wide range of geographic areas. Nonetheless, some limitations should also be considered. First, there is a risk of participation bias, i.e., those who did not take part may have different views which are not represented. British responders may have had a relatively high interest in the topic since they were either BEST3 collaborators [9] or connected to esophageal cancer consortia. Second, the general public’s perspective was not within the scope of this study and has been covered elsewhere [21]. Third, although we removed sorting data that was not categorized based on thematic similarity, some clusters still contained statements that did not optimally match the assigned label (e.g., the statement ‘There is no clear evidence on surgical prevention of reflux’ was grouped in the cluster ‘Patient education’). It is conceivable that participants who generated relatively few categories negatively impacted the quality of the data in terms of thematic similarity, by grouping statements together which are reasonably dissimilar in content [17]. Fourth, concept mapping is a mixed-methods approach primarily used to generate insights, it is not intended to test null-hypotheses regarding the relevance of themes. Average cluster ratings should therefore be interpreted in this light.

## Conclusions

Healthcare professionals’ identification and prioritization of issues associated with the acceptability of BE and EAC screening underscored the need to conduct prospective studies on screening benefits and harms and the appropriate target population. The screening modality needs to be minimally invasive and should have high specificity, sensitivity and public acceptance. GPs’ preference to play a role in the screening process needs to be assessed, and the readiness of endoscopy and pathology services to adopt a new screening tool needs to be evaluated. Lastly, the study has shown that potential implementation of BE or EAC screening requires country-specific regulations and commitments with insurance companies and governmental organizations to arrange screening.

### Supplementary Information

**Additional file 1.** Supplemental methods. The file contains detailed information on how multidimensional scaling and hierarchical cluster analysis were done.

**Additional file 2.** Profession types among participants and non-responders. The file contains participant characteristics of participants and non-responders.

**Additional file 3.** Final list of statements and clusters. The file contains a table with all statements generated during the brainstorm task, stratified by country and sorted by mean cluster/statement relevance rating. English translation of Dutch statements is included in this file.

## Data Availability

The dataset supporting the conclusions of this article can be accessed at 10.17026/dans-xmt-2dt7.

## Abbreviations

BE: Barrett’s esophagus
EAC: Esophageal adenocarcinoma
EMR: Endoscopic mucosal resection
ESD: Endoscopic submucosal dissection
GERD: Gastroesophageal reflux disease
GP: General practitioner
NHS: National Health Service
NL: Netherlands
RCT: Randomized controlled trial
TNE: Transnasal endoscopy
UK: United Kingdom
US: United States
