# Supplementary material for: Dutch, UK and US professionals’ perceptions of screening for Barrett’s esophagus and esophageal adenocarcinoma: a concept mapping study
Source: BMC Cancer. 2023 Nov 14;23:1111. doi: 10.1186/s12885-023-11583-x (PMC10647074; doi:10.1186/s12885-023-11583-x)
Supplement: Supplementary file 1 — Additional file 1. Supplemental methods. The file contains detailed information on how multidimensional scaling and hierarchical cluster analysis were done. [file 12885_2023_11583_MOESM1_ESM.docx]

**Additional file 1: Supplemental methods**

*Multidimensional scaling*

We created a similarity matrix showing the number of participants that sorted pairs of statements together. If two statements were considered thematically similar by participants, they were frequently sorted together and therefore positioned close to each other on a two-dimensional ‘point map’. The overall fit of the point map is described by a stress-value, which compares the obtained map to the similarity matrix that served as input. A stress-value between 0.21 and 0.37 represents sufficient fit, whereas a lower value within this interval is considered most optimal.^1^

*Hierarchical cluster analysis*

To separate the statements into non-overlapping clusters of related ideas, ‘cluster maps’ were created using a hierarchical cluster analysis. The software facilitates a maximum number of 15 clusters (i.e., the most specific content analysis). The number of clusters is then reduced iteratively until the default minimum number of 4 clusters is reached (i.e., the most general content analysis). One researcher (JS) systematically reviewed all generated cluster solutions by evaluating the content of each cluster and calculating the percentage of statements that were thematically similar. If the content within one cluster was thematically similar but not adequately represented by the label, another label was manually assigned (choosing from the labels that were formulated by participants during the sorting activity). For each country, we selected the cluster solution with the highest total percentage of thematic correspondence within each cluster. Additionally, mean cluster rating values were added to create cluster-rating concept maps. The cluster ratings are depicted as stacked layers on the maps, with more layers representing higher rating values for that cluster.

*Sorting task interface and specific wording*

The following screenshots depict the sorting task interface on the concept systems website, along with the corresponding instructions.


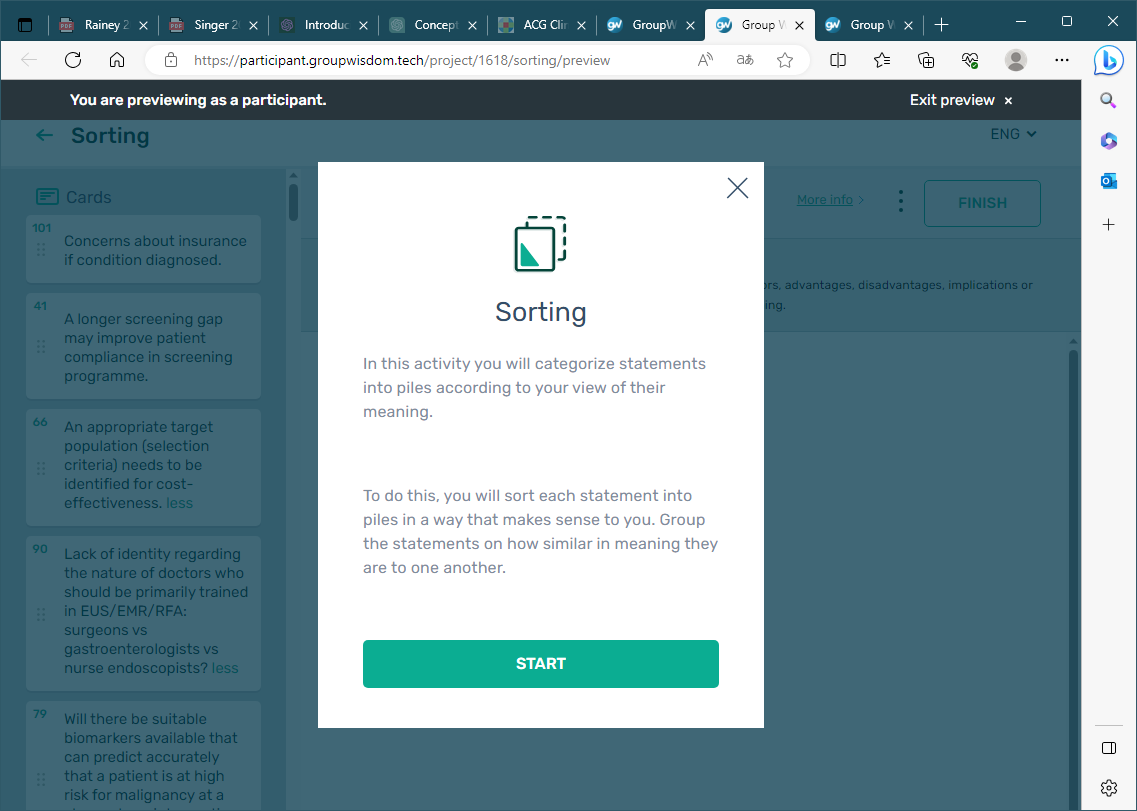


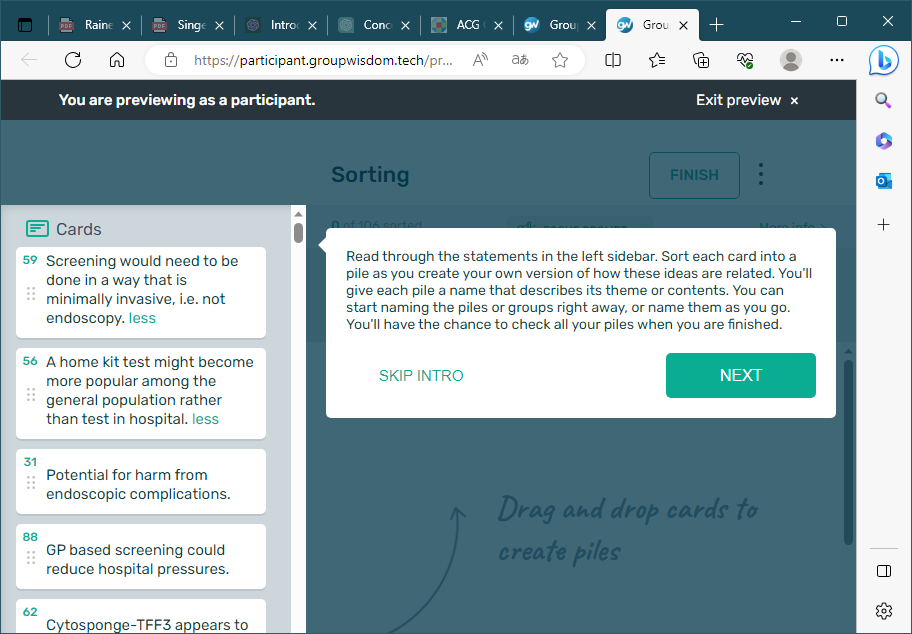


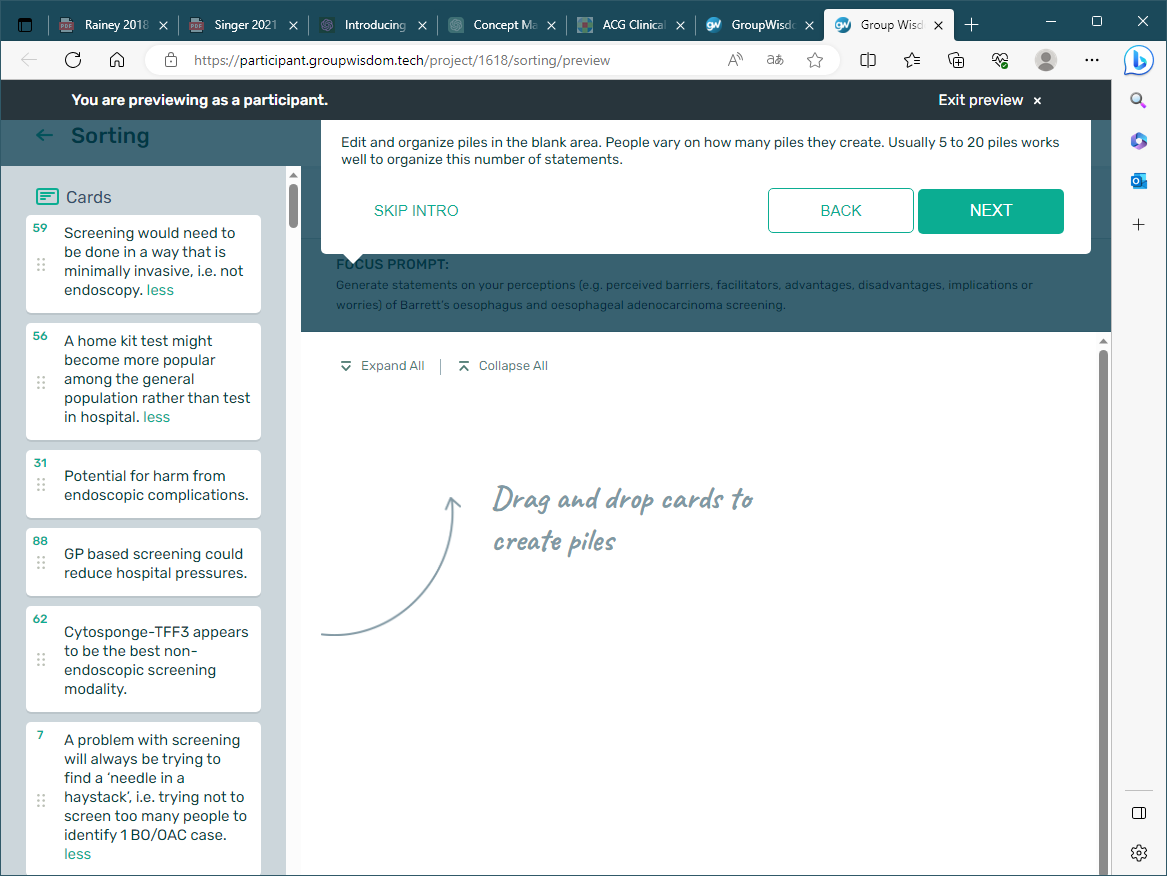


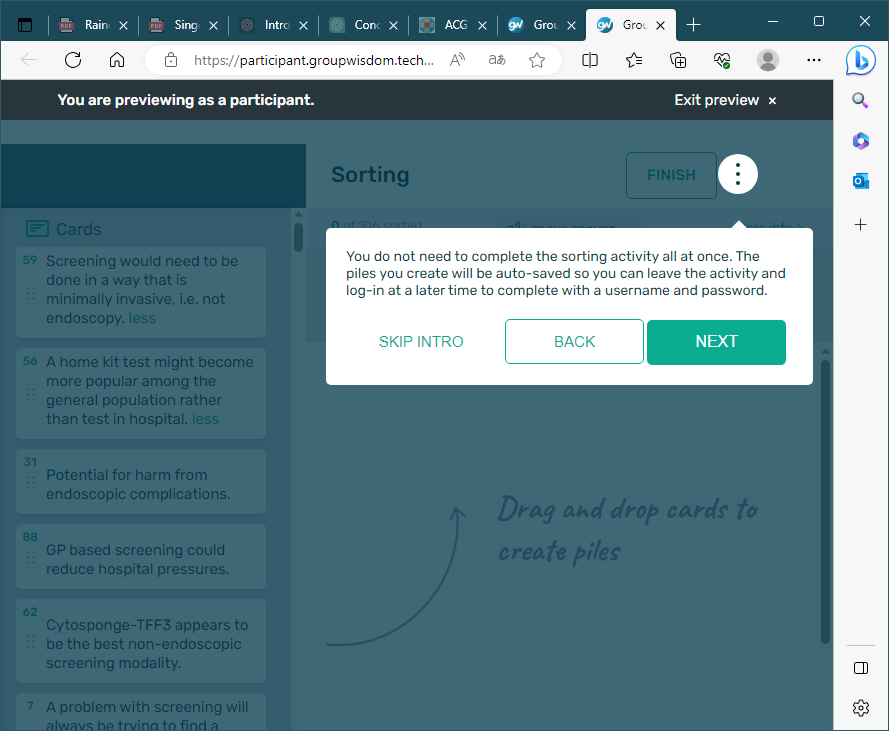


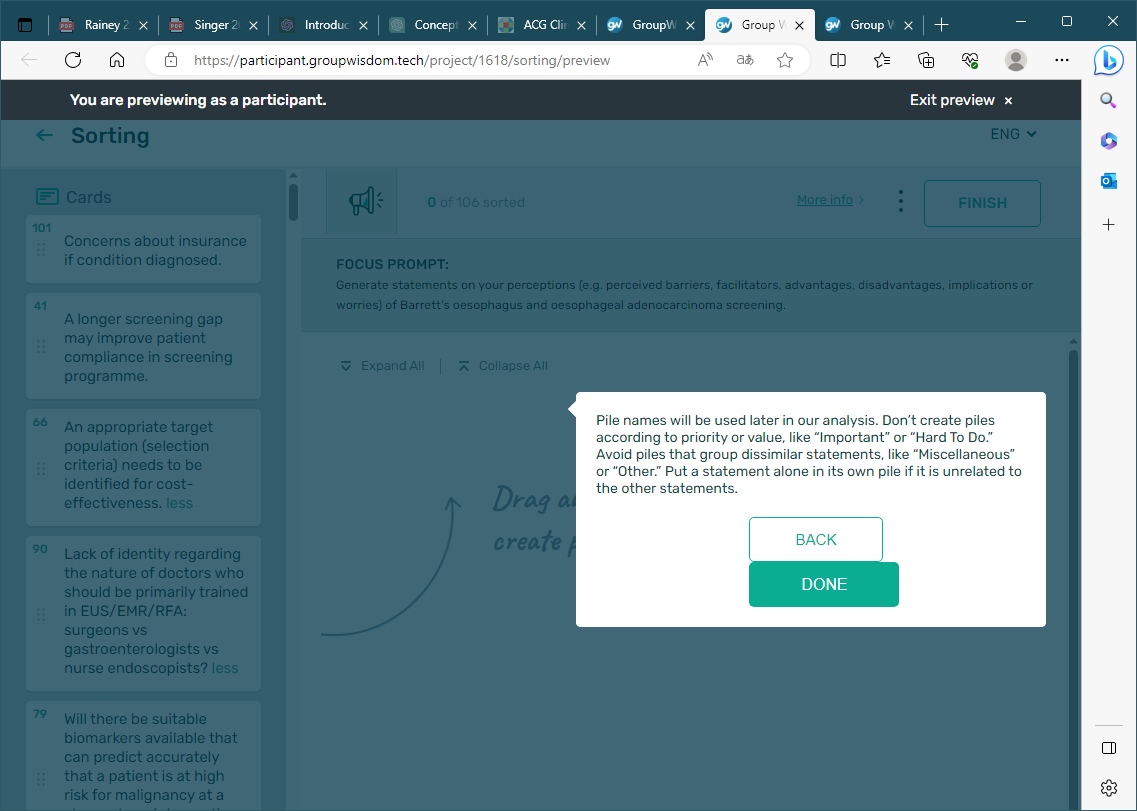


1. Concept Mapping for Planning and Evaluation. SAGE Publications, Inc.; 2007. <https://methods.sagepub.com/book/concept-mapping-for-planning-and-evaluation>
