# Supplementary material for: Dutch, UK and US professionals’ perceptions of screening for Barrett’s esophagus and esophageal adenocarcinoma: a concept mapping study
Source: BMC Cancer. 2023 Nov 14;23:1111. doi: 10.1186/s12885-023-11583-x (PMC10647074; doi:10.1186/s12885-023-11583-x)
Supplement: Supplementary file 2 — Additional file 2. Profession types among participants and non-responders. The file contains participant characteristics of participants and non-responders. [file 12885_2023_11583_MOESM2_ESM.docx]

**Additional file 2: Profession types among participants and non-responders.**

|  | **Netherlands** | | **United Kingdom** | | | | **United States** | |
| --- | --- | --- | --- | --- | --- | --- | --- | --- |
|  | Participants (n) | Non-responders (n) | | Participants (n) | Non-responders (n) | Participants (n) | | Non-responders (n) |
| *Invitation approach* | Personal invitation | | | General invitation | | Personal invitation | | |
| *Profession type* |  |  | |  | |  | |  |
| General practitioner (n) | 2 | 7 | | 3 | 73 | 1 | | 6 |
| Physician extender (n) | 2 | 0 | | 0 | 1 | 1 | | 2 |
| Gastroenterologist (n) | 8 | 5 | | 4 | 26 | 6 | | 1 |
| Oncologist (n) | 1 | 1 | | 2 | 4 | 3 | | 1 |
| Surgeon (n) | 2 | 1 | | 5 | 21 | 1 | | 0 |
| Radiotherapist (n) | 1 | 2 | | 0 | 0 | 0 | | 1 |
| Pathologist (n) | 3 | 1 | | 2 | 3 | 3 | | 1 |
| Radiologist (n) | 0 | 0 | | 2 | 1 | 0 | | 0 |
| Researchers (n) | 3 | 1 | | 2 | 4 | 1 | | 2 |
| Policy advisors (n) | 7 | 1 | | 0 | 1 | 2 | | 0 |
| **Total** | **29** | **19** | | **20** | **134** | **18** | | **14** |
