# Supplementary material for: Dutch, UK and US professionals’ perceptions of screening for Barrett’s esophagus and esophageal adenocarcinoma: a concept mapping study
Source: BMC Cancer. 2023 Nov 14;23:1111. doi: 10.1186/s12885-023-11583-x (PMC10647074; doi:10.1186/s12885-023-11583-x)
Supplement: Supplementary file 3 — Additional file 3. Final list of statements and clusters. The file contains a table with all statements generated during the brainstorm task, stratified by country and sorted by mean cluster/statement relevance rating. English translation of Dutch statements is included in this file. [file 12885_2023_11583_MOESM3_ESM.docx]

**Additional file 3: Clusters and statements, stratified by country and hierarchically sorted by mean relevance.**

| **Point on map** | **Cluster name/statement** | | | **No. of participants submitting the statement** | **Relevance rating (mean)** |
| --- | --- | --- | --- | --- | --- |
|  | | | |  |  |
| **The Netherlands** | | | |  |  |
|  | **Potential health benefits** | | |  | **6.51** |
| 105 | Detecting cancer at an early stage increases the likelihood of successful endoscopic treatment. | | | 4 | 8.15 |
| 104 | Early detection leads to a shift in the type of surgery (more EMRs/ESDs, fewer esophagectomies) and therefore fewer (major) complications. | | | 4 | 7.55 |
| 99 | Early detection of (precancerous) esophageal carcinoma will lead to fewer deaths. | | | 3 | 7.10 |
| 23 | Preventing cancer in the screening population may improve the quality of life of individuals. | | | 1 | 6.58 |
| 116 | The introduction of a screening programme will reduce the incidence of synchronous esophageal cancer metastasis. | | | 1 | 6.57 |
| 101 | May significantly reduce the incidence of esophageal adenocarcinoma. | | | 1 | 6.40 |
| 106 | Increased opportunity for curative treatment, i.e., resection | | | 1 | 6.29 |
| 103 | Preventive treatments for Barrett esophagus would be an advantage. | | | 1 | 6.20 |
| 109 | Screening may facilitate early detection of the disease. | | | 1 | 6.14 |
| 115 | The introduction of a screening programme will increase the quality of local excisions. | | | 1 | 4.15 |
|  |  | | |  |  |
|  | **Cost-effectiveness** | | |  | **6.33** |
| 51 | It is unclear whether screening for BE/EAC is cost-effective. | | | 4 | 7.62 |
| 50 | Costs are an important argument in introducing screening on a large scale. | | | 5 | 7.35 |
| 85 | Screening for Barrett's esophagus with a non-invasive test aimed at detecting Barrett's esophagus with (high-grade) dysplasia may be cost-effective. | | | 2 | 7.18 |
| 86 | A screening programme that specifically aims to detect precursors (Barrett esophagus) is not cost-effective. | | | 2 | 7.10 |
| 43 | The financial investment in screening will be recovered by preventing higher healthcare costs later on. | | | 1 | 6.91 |
| 49 | In my opinion, it is only cost-effective if there is a good pre-screening (analogous to, for example, the FiOBT). | | | 1 | 6.90 |
| 52 | Not cost-effective in case of population screening. | | | 1 | 6.80 |
| 48 | The cost-effectiveness of screening is related to the incidence of disease and the cost of the screening instrument. | | | 1 | 6.24 |
| 53 | The price of screening is high due to material costs and the deployment of manpower. | | | 2 | 6.20 |
| 44 | The cost of screening may be reduced by alternative/new techniques and alternative form of organization. | | | 2 | 6.10 |
| 47 | Expensive maintenance treatments for stage 1 esophageal cancer may limit cost-effectiveness of screening. | | | 1 | 5.05 |
| 54 | Reimbursement of screening and subsequent treatment by the same authority will increase support for screening. | | | 1 | 4.86 |
| 46 | Expressing benefit and risk in a cost-effectiveness ratio may be interesting from an accounting point of view, but hardly from a public health point of view. | | | 1 | 3.85 |
|  |  | | |  |  |
|  | **Design screening method** | | |  | **6.26** |
| 40 | The screening method should be highly specific to avoid having too many people undergoing endoscopy. | | | 4 | 8.00 |
| 41 | The screening method should be highly sensitive to avoid missing too many people. | | | 4 | 7.90 |
| 25 | Besides test characteristics such as specificity and sensitivity, the willingness of the public to undergo this test largely determines the success of a screening programme. | | | 4 | 7.89 |
| 42 | The actual ability of less invasive screening methods compared to oesophago-gastro-duodenoscopy to distinguish ill and non-ill persons is essential. | | | 5 | 7.76 |
| 22 | Patients have a preference for non-invasive screening tests. | | | 1 | 7.35 |
| 33 | Until there is a good non-invasive alternative to endoscopy, screening for esophageal cancer is not attractive. | | | 2 | 7.00 |
| 34 | We don't have enough experience with new screening methods for large-scale screening. | | | 3 | 6.90 |
| 36 | Esophagogastroduodenoscopy is not suitable for large-scale screening. | | | 2 | 6.71 |
| 27 | Individuals will be reluctant to participate because they find the test scary or burdensome. | | | 2 | 6.67 |
| 30 | Screening should be as non-invasive as possible, transnasal endoscopy is too invasive for individuals without symptoms in my opinion. | | | 4 | 6.30 |
| 39 | It is unclear whether individuals should be screened repeatedly. | | | 2 | 6.15 |
| 107 | An exit strategy from the screening in case of certain findings is crucial. | | | 1 | 6.14 |
| 65 | Applying screening techniques in primary care saves a large number of endoscopies. | | | 3 | 5.68 |
| 35 | Standard endoscopy is human-unfriendly. | | | 2 | 5.62 |
| 28 | Esophageal cancer is too little a problem among the public to generate enough enthusiasm to participate in screening. | | | 5 | 5.52 |
| 32 | A non-invasive test is harmful rather than useful because it lowers the threshold to start screening (even at low risk of cancer). | | | 1 | 4.20 |
| 74 | Patients with severe disease are unable to refuse esophageal cancer screening. | | | 1 | 3.43 |
| 31 | A non-invasive test, such as a breath test, reinforces the illusion of low-risk screening and is therefore harmful rather than helpful. | | | 1 | 3.71 |
|  |  | | |  |  |
|  | **Legitimacy** | | |  | **6.15** |
| 96 | Without mortality reduction (or other clinically relevant outcomes), detection of precursors (Barrett) and disease (esophageal cancer) only results in risk and even harm, but not in benefit! | | | 1 | 7.76 |
| 94 | Most patients with Barrett esophagus do not develop esophageal cancer or dysplasia. | | | 2 | 7.67 |
| 72 | Invasive screening for esophageal cancer among high-risk individuals should only be done if these people are well aware of the risks of both screening and refraining from screening. | | | 2 | 7.52 |
| 84 | As experience with this is limited, we do not know whether the advantages of screening outweigh the disadvantages. | | | 3 | 7.50 |
| 98 | Decrease in esophageal cancer mortality will be limited despite screening because asymptomatic patients with an increased risk of esophageal cancer are still missed. | | | 1 | 7.25 |
| 100 | Decrease in esophageal cancer incidence will be limited despite screening because asymptomatic patients with an increased risk of esophageal cancer are still missed. | | | 1 | 7.19 |
| 119 | Screening can be helpful to filter patients at risk from the large group with reflux complaints. | | | 1 | 7.00 |
| 120 | It is unclear whether the disease is sufficiently recognizable at an early stage. | | | 1 | 6.90 |
| 1 | The low incidence and low mean survival are barriers in screening for esophageal cancer | | | 1 | 6.76 |
| 90 | It is difficult to estimate the feasibility due to the knowledge gap of BE progression at a population level (interval cancers). | | | 4 | 6.62 |
| 89 | Insight into the incidence of BE in the screening population is insufficient, therefore pilot screening studies are needed. | | | 2 | 6.60 |
| 121 | The risk of Barrett's esophagus and esophageal cancer is accompanied by other comorbidities, which may limit the health benefits of screening. | | | 1 | 6.59 |
| 71 | Early cancer detection may result in a longer lifetime knowing you have cancer, but not a longer lifetime. | | | 1 | 6.55 |
| 93 | The prevalence of esophageal cancer is too low to initiate population screening. | | | 6 | 6.55 |
| 97 | Randomized research into mortality reduction effectiveness must be conducted before screening can be implemented. | | | 3 | 6.50 |
| 77 | Great benefit to few people. | | | 2 | 6.40 |
| 26 | Screening uptake is related to disease incidence, disease severity and test burden | | | 1 | 6.29 |
| 91 | The dismal prognosis of this cancer justifies early detection. | | | 3 | 6.29 |
| 118 | Preventing cancer in the screening population improves public health. | | | 1 | 6.24 |
| 92 | As the incidence of esophageal cancer is increasing rapidly in the Netherlands, the potential for screening has grown. | | | 1 | 6.10 |
| 2 | Even if it is detected through screening, people usually still die of esophageal cancer. | | | 1 | 5.74 |
| 73 | Screening always leads to risk and harm, sometimes to some good and rarely to more good than risk and harm. | | | 1 | 5.52 |
| 78 | It might be useful only for people who would die of esophageal cancer without population screening. | | | 1 | 5.00 |
| 80 | The more complex the benefit-risk ratio is, the higher the threshold for screening should be. | | | 1 | 5.00 |
| 82 | Adding an easily accessible screening test to an unfavorable benefit-risk ratio is like a fish trap with bait, or (for music lovers) like Hotel California: you can enter, but never leave. | | | 2 | 4.73 |
| 83 | Other findings or missing them can be a reason to refrain from screening for esophageal cancer, but should never be a reason to screen for 'just anything'! | | | 1 | 4.68 |
| 79 | The complexity of the benefit-risk ratio should be directly proportional to the complexity of the screening. | | | 1 | 4.40 |
| 76 | Benefit and risk are incomparable values | | | 1 | 4.09 |
| 81 | A relatively simple benefit-risk ratio does not justify low-threshold screening, because the screening is part of the benefit-risk ratio – a snake biting its tail. | | | 1 | 3.38 |
|  |  | | |  |  |
|  | **Capacity** | | |  | **5.92** |
| 29 | Adequate education of general practitioners and patients is essential. | | | 1 | 8.05 |
| 37 | Less-invasive screening methods (eNose, esosponge) may be suitable for large-scale screening. | | | 11 | 8.00 |
| 18 | Undergoing a screening test in primary care is more accessible for patients than endoscopy in the hospital. | | | 2 | 6.81 |
| 68 | The current endoscopy capacity is insufficient for the expected increase in Barrett esophagus surveillance. | | | 3 | 6.80 |
| 66 | A limiting factor may be the increase in work for gastroenterologists and pathologists. | | | 1 | 6.68 |
| 57 | A home testing kit (and not in the hospital or primary care) is preferable because of the burden on the health care system. | | | 2 | 6.65 |
| 69 | The national capacity for endoscopic treatment of early carcinomas is not sufficient to introduce a screening programme. | | | 1 | 6.35 |
| 75 | Shouldn't we focus on prevention (smoking/alcohol)? | | | 2 | 6.19 |
| 56 | Setting up a national system (such as the bowel cancer screening program, but a more specific group for this) ensures that screening for Barrett's esophagus/early carcinoma at the GP can be successful. | | |  | 6.00 |
| 67 | How are we going to handle the increase endoscopies? Do we have sufficient capacity in gastroenterologists or do we need to train more? We should avoid long waiting lists! | | | 4 | 5.81 |
| 38 | Using artificial intelligence for pill/sponge screening instrument histology assessment. | | | 1 | 5.65 |
| 63 | Screening at the GP will burden the healthcare system too much. | | | 2 | 5.50 |
| 58 | Applicable within 1.5 line care and the deployment of nursing specialists or physician assistants. | | | 2 | 5.45 |
| 64 | Esophageal cancer screening should be performed in primary care. | | | 2 | 5.00 |
| 59 | Treating a Barret's esophagus is well done in general practice. | | | 1 | 3.30 |
| 123 | Ownership of screening within primary care will result in referral delay. | | |  | 2.52 |
|  |  | | |  |  |
|  | **Harms of screening** | | |  | **5.80** |
| 113 | Detection of Barrett's esophagus without dysplasia leads to overdiagnosis of patients who would never have developed esophageal cancer in their lives. | | | 7 | 8.10 |
| 112 | The risk of overtreatment which results in more complications. | | | 1 | 7.57 |
| 111 | Detection of Barrett's esophagus without dysplasia results in a lifetime of largely unnecessary surveillance endoscopies. | | | 2 | 6.71 |
| 19 | Screening for esophageal cancer by detecting Barrett's esophagus often leads to false reassurance. | | | 1 | 6.24 |
| 70 | Disadvantages: complications of biopsies. | | | 1 | 5.86 |
| 110 | A barrier to detecting more early stages is that imaging and endoscopic techniques are not yet able to properly distinguish between T1 and T2 carcinomas, thus limiting good treatment advice (surgery vs endoscopic resection, pre-treatment in case of surgery). | | | 1 | 5.55 |
| 102 | An esophagectomy is quite disabling. | | | 1 | 5.35 |
| 117 | The introduction of a screening programme will increase the quality of pathological assessment. | | | 1 | 5.20 |
| 21 | Screening for Barrett's esophagus will result in anxiety among reflux patients. | | | 3 | 4.95 |
| 88 | Patients with Barrett's esophagus use proton pump inhibitors for a lifetime. Side effects? Cost? Medicalization? | | | 2 | 4.81 |
| 20 | Screening for Barrett's esophagus will be reassuring for reflux patients. | | | 2 | 4.75 |
| 114 | The introduction of a screening programme will increase the quality of endoscopic assessment. | | | 1 | 4.40 |
|  |  | | |  |  |
|  | **General considerations** | | |  | **5.50** |
| 108 | Should also go hand in hand with more effective surveillance in patients with known Barrett (less, better, selection of a population at risk, reduction of costs). | | | 2 | 6.90 |
| 122 | Don't start considering screening for esophageal cancer until Wilson & Junger’s 10 criteria are genuinely fulfilled, it's not a tick list. | | | 1 | 6.90 |
| 45 | Screening for Barrett's esophagus/early carcinoma in primary care will save cost compared to endoscopy in the hospital. | | | 3 | 6.59 |
| 95 | Most esophageal carcinomas are discovered in patients not known to have Barrett's esophagus… | | | 1 | 6.30 |
| 55 | Given the freedom for individuals to choose a health insurer, including switching to another insurer, the screening will need to be publicly funded. | | | 1 | 5.70 |
| 87 | Squamous cell carcinoma should also be included in a screening program. | | | 1 | 4.60 |
| 3 | Screening for esophageal cancer by detecting Barret's esophagus is just health care on indication. | | | 1 | 3.85 |
| 61 | Patients with Barrett Esophagus can come to the GP when they have symptoms, so why should we screen? | | | 1 | 2.95 |
|  |  | | |  |  |
|  | **Target population** | | |  | **5.46** |
| 5 | It is unclear whether a relevant risk group can be defined for this disease. | | | 3 | 7.70 |
| 6 | A good population-based study should be carried out to determine which risk groups are relevant. | | | 5 | 7.57 |
| 16 | A clear guideline should be written about who is eligible for esophageal cancer screening. | | | 1 | 7.57 |
| 11 | Defining the age range for screening is essential. | | | 2 | 6.76 |
| 12 | Population groups at risk (alcoholism) are probably difficult to reach. | | | 1 | 6.05 |
| 4 | Screening based on risk factors (age > 50, overweight, white race, etc?) | | | 6 | 5.95 |
| 24 | Patients with GERD are open to esophageal cancer screening. | | | 2 | 5.80 |
| 8 | Should be offered to people with chronic reflux symptoms. | | | 3 | 5.48 |
| 62 | Selecting the population at risk is complicated and requires training for doctors or other healthcare personnel. | | | 2 | 5.48 |
| 9 | For all patients with more than 10 years of PPI use and no previous endoscopy. | | | 3 | 5.05 |
| 13 | The inclusion criterion ‘white male’ is a form of ethnic discrimination, which is politically questionable. | | | 1 | 4.71 |
| 7 | The narrow definition of the screening population may result in non-eligible individuals demanding a screening test (like breast cancer screening at an older age). | | | 1 | 4.67 |
| 60 | The GP should select patients eligible for screening. | | | 2 | 4.50 |
| 15 | Obesity is a risk factor, and waist-hip ratio is more relevant than BMI. | | | 1 | 4.00 |
| 17 | Not appropriate in case of dysphagia, this warrants direct referral for endoscopy. | | | 1 | 3.90 |
| 14 | Risk population: using data on alcohol and nicotine abuse in hospital information systems. | | | 1 | 3.81 |
| 10 | Combining BE/EAC screening with other screening programs (breast of colorectal cancer), possibly only for individuals with a high BMI or alcohol/nicotine abuse. | | | 1 | 3.70 |
|  |  | | |  |  |
|  |  | | |  |  |
|  |  | | |  |  |
|  |  | | |  |  |
|  |  | | |  |  |
|  |  | | |  |  |
|  |  | | |  |  |
|  |  | | |  |  |
| **UK** |  | | |  |  |
| **Point on map** | **Cluster name/statement** | | | **No. of participants submitting the statement** | **Relevance rating (mean)** |
|  | | **Screening population** | |  | **8.01** |
| 66 | | An appropriate target population (selection criteria) needs to be identified for effectiveness. | | 5 | 8.41 |
| 83 | | There is a growing population at risk of BO and OAC in the UK due to the increase in risk factors such as obesity, reflux etc therefore an increased need for screening. | | 1 | 8.24 |
| 84 | | Using some sort of risk prediction tool to identify a ‘high risk’ group in which screening could be conducted. | | 1 | 8.12 |
| 85 | | Offering screening to participants with higher pre-test probability than general population would be a facilitator. | | 6 | 7.71 |
| 60 | | What are the sensitivities and specificities of the alternatives to endoscopic screening? | | 5 | 7.59 |
|  | |  | |  |  |
|  | | **Screening modality** | |  | **7.05** |
| 57 | | Screening tool should be easily administered. | | 2 | 8.63 |
| 63 | | The availability of less invasive techniques should facilitate screening in this condition. | | 4 | 7.94 |
| 59 | | Screening would need to be done in a way that is minimally invasive, i.e. not endoscopy. | | 4 | 7.71 |
| 58 | | No need for sedation would facilitate screening. | | 1 | 7.69 |
| 62 | | Cytosponge-TFF3 appears to be the best non-endoscopic screening modality. | | 2 | 7.59 |
| 56 | | A home kit test might become more popular among the general population rather than test in hospital. | | 3 | 7.59 |
| 61 | | A blood / stool / saliva test would be ideal. | | 2 | 7.47 |
| 23 | | Confounders need to be clearly identified and pointed out to reduce false negatives. | | 1 | 7.18 |
| 22 | | Technologies in early detection / artificial intelligence could be facilitators to helping achieve a minimally invasive method of screening. | | 1 | 6.94 |
| 41 | | A longer screening gap may improve patient compliance in screening programme. | | 1 | 6.24 |
| 76 | | The test should be combined with gastric cancer screening/screening for gastric atrophy (e.g. pepsinogen blood test, volatile markers etc). | | 1 | 5.41 |
| 14 | | The test should include oesophageal squamous cell carcinoma screening. | | 1 | 4.41 |
|  | |  | |  |  |
|  | | **Recommended service organisation** | |  | **6.97** |
| 43 | | Clear pathways need to be defined for the onward referral process. | | 1 | 8.41 |
| 21 | | Effective pathology service required. | | 1 | 8.38 |
| 20 | | Easy access in the community would facilitate OAC screening. | | 2 | 8.29 |
| 73 | | Who will be the main professional group/workforce to deliver screening programme for BO and OAC? | | 1 | 8.06 |
| 40 | | Increase in diagnostic endoscopy as result of screening needs to be modelled and factored into plans to allow appropriate renumeration at Trust level. | | 1 | 8.00 |
| 93 | | If the screening tool is GP based, will the hospitals be able to provide the endoscopy services to confirm the diagnoses? | | 2 | 7.94 |
| 46 | | A national screening roll-out should be accompanied by a systematic data collection for service and epidemiological research. | | 1 | 7.88 |
| 42 | | Time points for testing need to be defined (one off vs regular intervals). | | 3 | 7.82 |
| 98 | | Good patient communication required. | | 3 | 7.65 |
| 50 | | Screening within the NHS (UK) should be organised by national roll-out. | | 1 | 7.59 |
| 49 | | A 2-step program with specialist referral for OGD remains necessary as endoscopic assessment plus biopsies remains the (invasive) diagnostic gold standard. | | 2 | 7.41 |
| 64 | | The recent major pressures on endoscopy workload may paradoxically prove helpful in facilitating this screening by leading to wider acceptance of the Cytosponge-TFF3. | | 1 | 7.35 |
| 53 | | If GP was facilitated / funded / staffed it might work and be great for patients. | | 1 | 7.06 |
| 47 | | Results to be conveyed to GP efficiently. | | 4 | 6.94 |
| 77 | | How can it be joined up with other screening programmes? | | 1 | 6.82 |
| 54 | | In the UK, testing should be facilitated at GP surgeries if at home testing is not feasible. | | 2 | 6.76 |
| 48 | | Read-out of any test should be ubiquitously feasible, not only in a few centralised labs. | | 1 | 6.76 |
| 19 | | This pre-screening must not distract from direct specialist referral in case of alarm symptoms. | | 1 | 6.71 |
| 75 | | Could the test be linked with the national bowel cancer screening program to increase efficacy and participation rate? | | 1 | 6.69 |
| 88 | | GP based screening could reduce hospital pressures. | | 1 | 6.35 |
| 71 | | Multidisciplinary team required to support the screening service and discuss it with patients. | | 1 | 6.24 |
| 45 | | A uniform approach should be used in different countries to allow international comparison of results. | | 1 | 6.06 |
| 55 | | Mobile units would work best in rural areas. | | 2 | 6.00 |
| 70 | | It would need an independent service to provide this as screening, not in general practice. | | 2 | 4.94 |
| 39 | | Screening should take place with admin separate from endoscopy booking (as there is concern that screening will be seen as extension of endoscopy task). | | 1 | 4.71 |
| 74 | | OAC screening should be performed as part of routine care by a health care professional known to the patient. | | 4 | 4.47 |
|  | |  | |  |  |
|  | | **Clinical Need** | |  | **6.89** |
| 35 | | The attractive theoretical benefit of detecting more cases of BO and applying endoscopic therapy to reduce OAC incidence. | | 5 | 7.50 |
| 37 | | Strong potential to reduce mortality from OAC in areas with high incidence of disease. | | 8 | 7.47 |
| 33 | | Knowledge that patients with OAC who have BO diagnosed prior to their cancer have improved survival outcomes could be a facilitator. | | 1 | 7.06 |
| 38 | | Less invasive treatment will result in fewer treatment related morbidity. | | 4 | 7.00 |
| 69 | | Reduces healthcare cost. | | 1 | 6.53 |
| 81 | | Should males be screened regardless of reflux symptoms given the high prevalence of the condition in men? | | 1 | 6.47 |
| 13 | | Regional and genetic differences amongst certain communities for eg: Black, Asian and Minority Ethnic people. | | 1 | 6.24 |
|  | |  | |  |  |
|  | | **Patient education** | |  | **6.81** |
| 24 | | How does the stress of having a “pre-malignant lesion” effect the quality of life of patients with BO? | | 1 | 7.53 |
| 104 | | There is need for patients to understand the implication of a positive screening test…. What would follow? | | 2 | 7.41 |
| 106 | | Awareness and understanding of the disease among the general population … what is the perception? At least my family members have no understanding of this disease. | | 1 | 7.00 |
| 105 | | Patient education regarding the link between chronic reflux and OAC remains poor, limiting early diagnosis. | | 2 | 6.76 |
| 36 | | Would impact positively on patients who are anxious about symptoms or family history. | | 2 | 6.35 |
| 8 | | There is no clear evidence on surgical prevention of reflux. | | 2 | 5.82 |
|  | |  | |  |  |
|  | | **Natural history unknowns** | |  | **6.75** |
| 79 | | Will there be suitable biomarkers available that can predict accurately that a patient is at high risk for malignancy at a stage where intervention is possible? | | 6 | 7.59 |
| 12 | | Do we truly know the natural history of all stages of dysplastic lesion and mutated normal epithelium and can we accurately say which will progress to malignancy? | | 2 | 7.41 |
| 78 | | If you detect BO or low grade dysplasia, what happens to these patients? | | 1 | 7.35 |
| 4 | | Will the risk of malignancy be the same in screen detected BO versus non screen detected BO? | | 1 | 6.82 |
| 32 | | Don’t we still risk missing the more aggressive cancers that occur rapidly and are these the ones most associated with poor outcomes? | | 5 | 6.06 |
| 9 | | What to do with very short segments? | | 1 | 5.24 |
|  | |  | |  |  |
|  | | **Roll-out concerns** | |  | **6.62** |
| 67 | | How will a screening programme for BO and OAC be funded? | | 1 | 8.65 |
| 91 | | Limit of hospital resources (time, space and staff) to provide endoscopy services. | | 4 | 8.12 |
| 97 | | Will the uptake/tolerability of the screening be high enough to have an impact on OAC detection and ultimately cancer survival? | | 5 | 8.00 |
| 72 | | GP surgeries and most NHS services already under pressure to deliver services. | | 2 | 7.94 |
| 89 | | Screening would generate a high number of patients with BO that would increase surveillance workload into an already stretched system. | | 6 | 7.88 |
| 87 | | The lack of resources and training and additional set-up costs for cytosponge and ultrathin endoscopy are barriers to setting up screening. | | 3 | 7.76 |
| 16 | | Concern that screening would not reach the hardest-to-reach population groups at most risk. | | 2 | 7.35 |
| 68 | | In the context of the limited gains seen within lung cancer screening and colorectal screening, will it be cost-effective in a rationed heath care setting? | | 1 | 7.29 |
| 1 | | Buyers should not mandate population-level introduction of Barrett’s screening until mortality benefit Barrett’s screening has been tested and documented. | | 1 | 7.18 |
| 44 | | Increased numbers of patients might be referred for further investigations as a result of false positives screening tests. | | 1 | 7.18 |
| 92 | | Lack of trained specialists to perform detailed BO mapping. | | 6 | 6.76 |
| 65 | | One disadvantage of screening is that it is expensive. | | 4 | 6.71 |
| 86 | | The workload pressures to catch up post-COVID would restrict starting such a new screening programme. | | 4 | 6.71 |
| 18 | | Other screening programmes may have priority. | | 1 | 5.94 |
| 17 | | Lack of benefit to the general population. | | 1 | 5.71 |
| 95 | | Unfamiliarity with a new screening programme. | | 1 | 5.65 |
| 94 | | Lack of enthusiasm especially by surgeons in performing meaningful and thorough endoscopic surveillance of BO. | | 1 | 5.59 |
| 100 | | Would patients also want an endoscopy for reassurance? | | 1 | 5.35 |
| 28 | | OAC screening is too invasive. | | 1 | 5.24 |
| 51 | | The test will take too much time in my surgery. | | 3 | 5.12 |
| 52 | | Patients' travel time. | | 1 | 4.94 |
| 90 | | Lack of identity regarding the nature of doctors who should be primarily trained in EUS/EMR/RFA: surgeons vs gastroenterologists vs nurse endoscopists? | | 1 | 4.47 |
|  | |  | |  |  |
|  | |  | |  |  |
|  | | **Clinical effectiveness concerns** | |  | **6.45** |
| 10 | | Are there appropriate, cost effective and low risk interventions for early stages of disease? | | 1 | 8.06 |
| 34 | | Screening for BO patients alone will not identify all of the cancer cases (many OAC cases diagnosed without a prior diagnosis of BO). | | 1 | 6.82 |
| 82 | | It is difficult to select the population to target for screening since about 40% of patients with OAC denies previous history of reflux. | | 1 | 6.76 |
| 3 | | Lack of documented mortality benefit Barrett’s surveillance will translate to a lack of documented benefit Barrett’s screening. | | 1 | 6.71 |
| 30 | | How do we cope with other pathology detected, gastritis, ulcers, etc.? | | 1 | 6.59 |
| 6 | | BO has malignant potential but still lifetime risk is low. | | 1 | 6.59 |
| 102 | | How uncomfortable is the proposed screening? | | 3 | 6.53 |
| 80 | | Lack of simple methodologies to identify low risk patients who require no further surveillance. | | 1 | 6.29 |
| 7 | | A problem with screening will always be trying to find a ‘needle in a haystack’, i.e. trying not to screen too many people to identify 1 BO/OAC case. | | 4 | 6.18 |
| 96 | | Lack of clinician's understanding of why patient would be at risk. | | 2 | 5.76 |
| 5 | | OAC is more aggressive than other cancers that have been successfully screened. | | 1 | 5.59 |
| 11 | | Diagnosis can be unclear – histologic vs endoscopic findings. | | 1 | 5.47 |
|  | |  | |  |  |
|  | | **Impact on individual** | |  | **6.16** |
| 15 | | The absolute and relative costs involved without clear proven benefit of cost-effectiveness or effects on mortality. | | 1 | 7.41 |
| 27 | | Detects cancers and pre-cancerous conditions that may never lead to symptoms and illness and death (overdiagnosis). | | 6 | 6.76 |
| 31 | | Potential for harm from endoscopic complications. | | 1 | 6.76 |
| 26 | | Increasing anxiety in screening participants awaiting results. | | 2 | 6.59 |
| 29 | | Screening will lead to overtreatment of BO. | | 1 | 6.53 |
| 25 | | Increased anxiety in the screened population, exacerbated by the lack of clear pharmacological or lifestyle interventions that are effective. | | 5 | 6.47 |
| 103 | | Patient expectations of procedure involved: “ it’s like an endoscopy”, “I will have to go to the hospital”. | | 1 | 5.65 |
| 2 | | Barrett’s screening will provoke unnecessary oesophagectomies of patients overdiagnosed with non-lethal early oesophageal cancers. | | 1 | 5.41 |
| 101 | | Concerns about insurance if condition diagnosed. | | 1 | 5.18 |
| 99 | | Patient: “I will need sedation for this type of thing!” | | 1 | 4.82 |
| **US** | |  | |  |  |
| **Point on map** | | **Cluster name/statement** | |  | **Relevance rating (mean)** |
|  | | | **Impact of screening?** |  | **7.57** |
| 65 | | | Does screening for BE/EAC impact EAC mortality? | 2 | 9.00 |
| 64 | | | Does screening for BE/EAC impact EAC incidence? | 4 | 8.86 |
| 30 | | | We need better risk stratification tools (epidemiological, biomarkers etc.) to identify patients with non-dysplastic BE who are likely to progress to EAC | 4 | 8.29 |
| 58 | | | Most patients with BE will never develop EAC | 2 | 8.21 |
| 66 | | | Early detection of EAC | 2 | 8.21 |
| 27 | | | Even if we screen those with greatest likelihood to have BE (Caucasian males over age 50 with BMI over 25, smokers, long term reflux symptoms, long term PPI therapy, FMH, known hiatal hernia), there are still those with BE who develop EAC who do not fit any of these demographics | 1 | 8.14 |
| 59 | | | Although EAC is a low incidence cancer in the US, its high morbidity may justify screening | 1 | 7.79 |
| 56 | | | Warning symptoms/signs to screen for EAC occur at a late stage | 2 | 7.71 |
| 67 | | | Opportunity in precursor/stepwise nature of the disease | 1 | 7.36 |
| 4 | | | Preventing BE will prevent EAC | 1 | 7.14 |
| 15 | | | Providers are generally not aware of treatment options for BE beyond acid suppression | 1 | 6.57 |
| 51 | | | Is EAC a disease of sufficient burden to merit all of these efforts? | 1 | 6.36 |
| 6 | | | The solution to the problem of preventing EAC is not screening for BE; it is earlier diagnosis of GERD and control of the early progression of GERD aimed at preventing BE. Intense study of the feasibility of this new screening method should happen. | 1 | 4.79 |
|  | | |  |  |  |
|  | | | **Operationalization and partnership** |  | **7.21** |
| 33 | | | Identification of which patients would most benefit from screening should be improved | 4 | 8.50 |
| 32 | | | Acceptable threshold for risk of harboring BE at which screening is justified? | 1 | 8.21 |
| 79 | | | Gastroenterology societies could better liaise with internal medicine and family practice societies to increase awareness of BE/EAC | 1 | 8.21 |
| 29 | | | Can we develop an app or risk prediction tool that is easy to apply in clinical practice? | 1 | 8.14 |
| 9 | | | When should screening be done? (office visit, at time of colonoscopy, as part of FIT test administration) | 1 | 8.07 |
| 57 | | | Common lack of symptoms for BE/EAC; patient nor the care provider feel they meet criteria for screening | 1 | 8.00 |
| 61 | | | The low correlation between symptomatic GERD and BE is a barrier to screening | 1 | 8.00 |
| 46 | | | How do we empower primary care providers to perform screening? | 5 | 7.93 |
| 47 | | | How do we empower primary care providers to identify patients for screening? | 5 | 7.71 |
| 31 | | | Development of risks assessment tools that do not consider GERD are important | 3 | 7.64 |
| 49 | | | Primary care providers are too busy to discuss esophageal cancer risks and screening | 1 | 7.57 |
| 48 | | | Primary care providers are unaware of the emerging less invasive tools to screen for BE | 2 | 7.50 |
| 50 | | | Is BE a disease of sufficient burden to merit all of these efforts? | 1 | 7.43 |
| 11 | | | How often should screening take place? | 2 | 7.29 |
| 5 | | | We should recognize the present errors in the diagnosis of GERD (this is easy and without cost) | 1 | 6.64 |
| 8 | | | Combining BE screening with colorectal cancer screening could facilitate uptake | 1 | 6.64 |
| 36 | | | Who will take ownership of screening? | 1 | 6.57 |
| 37 | | | Gastroenterologists may facilitate screening | 1 | 6.43 |
| 93 | | | Cancer screening overload | 1 | 6.36 |
| 28 | | | Artificial intelligence or machine learning to identify candidates for screening | 1 | 6.21 |
| 42 | | | Lack of appreciation of how relatively easy it is to add a screening endoscopy to a screening colonoscopy for patients at risk for BE/EAC | 1 | 5.43 |
| 75 | | | Screening for Barrett’s is more accepted in Asian countries due to screening for gastric cancer in these countries | 1 | 4.14 |
|  | | |  |  |  |
|  | | | **Resources and reimbursement** |  | **6.73** |
| 101 | | | Universal BE screening is not cost-effective | 1 | 8.00 |
| 96 | | | Convincing insurance companies that screening for BE once in a lifetime should be a covered benefit paid in full (like it is done for colonoscopy for colon cancer screening) | 1 | 7.93 |
| 74 | | | New screening tools may soon be more widely available | 1 | 7.86 |
| 100 | | | Surveillance of BE is not cost-effective | 1 | 7.86 |
| 71 | | | What is optimal technology for screening or shall we choose from a menu of options? | 1 | 7.71 |
| 40 | | | How will we avoid low value care when screening large volumes? | 1 | 7.50 |
| 76 | | | EGD costs money to most patients of non-Medicare age (avoidance) | 2 | 7.43 |
| 1 | | | Who will pay for it? (technology, physician time, ancillary health time, assay performance and report generation) | 1 | 7.29 |
| 73 | | | Invasiveness of testing | 5 | 7.07 |
| 54 | | | Screening is complicated | 1 | 6.71 |
| 77 | | | Will patients accept any unsedated approach to screening? | 1 | 6.62 |
| 95 | | | Access to testing | 1 | 6.57 |
| 38 | | | Who is in charge of follow-up? | 1 | 6.43 |
| 45 | | | Competition for EGD resources from other indications | 2 | 6.43 |
| 99 | | | Upper endoscopy with propofol anesthesia is too costly | 3 | 5.86 |
| 10 | | | What infrastructure in the office is needed? | 1 | 5.79 |
| 97 | | | Precertification from insurance companies are required to approve EGD for screening of BE which takes time and soft costs to the practice | 1 | 5.79 |
| 41 | | | Endoscopy based screening is overused in the US | 1 | 5.71 |
| 98 | | | Reimbursement for colonoscopy is greater than upper endoscopy sadly perhaps skewing gastroenterologists to do more of the former | 1 | 5.21 |
| 43 | | | It currently takes months to obtain an appointment to undergo routine endoscopy | 2 | 4.86 |
|  | | |  |  |  |
|  | | | **Barrett’s esophagus surveillance issues** |  | **6.65** |
| 19 | | | Variability in quality of endoscopic practices | 1 | 8.36 |
| 68 | | | Being able to diagnose BE without an EGD like the electronic nose, blood tests or device (Esocheck, cytosponge) would help greatly | 1 | 8.07 |
| 26 | | | Many endoscopists sample BE insufficiently to detect precursor lesions | 1 | 7.71 |
| 72 | | | For noninvasive technologies how do we address false positives and false negatives | 1 | 7.50 |
| 16 | | | Once a diagnosis of BE is made, providers are uncertain how often the patients need to be surveyed | 2 | 7.50 |
| 13 | | | Lack of confidence among general endoscopists in identifying and biopting abnormal Z-lines | 2 | 7.36 |
| 34 | | | How will we deal with the potential overdiagnosis of BE? | 1 | 7.36 |
| 23 | | | Poor interobserver variability in the pathologist assessment of dysplasia in BE | 1 | 7.21 |
| 53 | | | Patients are sometimes provided a BE diagnosis and then lost to follow-up | 1 | 7.14 |
| 60 | | | Knowledge gaps in factors related to progression from BE to EAC | 1 | 6.86 |
| 24 | | | Pathology societies could develop standardized reporting for biopsies of the columnar esophagus | 1 | 6.79 |
| 39 | | | Who will interpret cytology/histology? | 1 | 6.79 |
| 22 | | | Barriers to obtaining an expert pathologist's second opinion (relationship with the general pathologist, administrative work and sample transportation cost) | 1 | 6.57 |
| 25 | | | A system akin to colorectal “adenoma detection rate” could be developed for detection of BE and associated lesions | 1 | 6.29 |
| 69 | | | Delayed/lack of clinical adoption of less invasive biomarkers of BE/EAC which have been studied and are now available is an impediment | 1 | 6.29 |
| 55 | | | Will it lead to widespread misdiagnosis of BE; normal z line epidemic? | 1 | 6.07 |
| 70 | | | EGD seems to be a reasonably reliable screening tool for EAC | 1 | 5.79 |
| 17 | | | Visual BE diagnosis without the need for biopsy would be very beneficial | 1 | 5.43 |
| 44 | | | BE surveillance requires extensive work on part of the endoscopist relative to other endoscopy indications | 1 | 5.36 |
| 3 | | | There is justification by evidence to immediately validate new pathologic definitions of GERD. There is justification for agreement that the proximal limit of rugal folds is not a reliable definition of the GEJ at endoscopy. Locating the GEJ at endoscopy is unreliable. | 1 | 4.79 |
| 18 | | | Non-goblet cell BE exists and complicates diagnosis in some cases | 1 | 4.43 |
|  | | |  |  |  |
|  | | | **Public awareness and communication** |  | **6.51** |
| 62 | | | Heartburn is a common symptom and people can self-medicate for months to years without seeking help | 3 | 7.79 |
| 63 | | | The majority of patients who present with EAC do not ever recall experiencing a reflux symptom | 3 | 7.43 |
| 86 | | | Public awareness about BE and screening options is low | 3 | 6.93 |
| 78 | | | Public education on lifestyle modifications that reduce likelihood of developing BE/EAC (also for PCP) | 2 | 6.64 |
| 7 | | | Provide written information (pamphlet) to patients at risk for BE (should be distributed by PCPs and gastroenterologists) | 1 | 6.57 |
| 83 | | | Using social and website media to impress upon the population the importance of BE screening | 1 | 6.43 |
| 85 | | | Public awareness about the risk of BE progressing to high grade dysplasia or cancer is low | 1 | 6.29 |
| 84 | | | Improving public awareness by appropriate labeling on bottles of over the counter PPI and in advertising by makers of these compounds | 2 | 5.79 |
| 35 | | | The importance of esophageal cancer is underscored by publicity and funding | 1 | 5.64 |
| 94 | | | Screening based on established risk factors such as male sex and white race could exacerbate health disparities | 1 | 5.64 |
|  | | |  |  |  |
|  | | | **Endotherapy** |  | **6.07** |
| 12 | | | We have the tools to safely and effectively eradicate early EAC (intra-mucosal, stage 1a) | 1 | 8.07 |
| 87 | | | Can early intervention impact on overall quality of life? | 1 | 7.43 |
| 88 | | | A diagnosis of a pre-malignant condition, without intervention to treat it, often results in use of unproven interventions | 3 | 6.36 |
| 2 | | | Will it help address healthcare disparities (impact minority patients)? | 1 | 6.33 |
| 52 | | | Many cancer registries combine ESCC and EAC; this is a barrier to understanding the true impact of BE screening programs | 1 | 6.14 |
| 21 | | | Ablation techniques improve with diminution of morbidity (strictures); opportunity to ablate non-dysplastic Barrett mucosa | 2 | 5.29 |
| 20 | | | Standardization of ablation techniques and their indications could be augmented | 1 | 5.21 |
| 14 | | | Treatments for esophageal cancer are so poor that it does not make sense to screen for it | 1 | 3.86 |
|  | | |  |  |  |
|  | | | **Patient fear** |  | **5.35** |
| 89 | | | How will we reassure patients diagnosed with very low risk BE? | 1 | 7.14 |
| 90 | | | The mere diagnosis of Barrett’s causes significant anxiety amongst patients | 1 | 6.79 |
| 92 | | | Difficulty with patient compliance | 1 | 6.07 |
| 80 | | | Patient perception of risk and actual data are non-congruent (risk overestimation) | 1 | 5.50 |
| 91 | | | Anxiety around testing for patients | 4 | 5.36 |
| 82 | | | Patients have friends or relatives who have suffered from esophageal cancer therapy and/or death and are afraid of screening | 1 | 3.64 |
| 81 | | | Patients hear worst-case stories from friends and relatives about endoscopic procedures | 1 | 2.93 |

| **Translation of Dutch statements** |  |
| --- | --- |
|  |  |
| **English translation** | **Original Dutch statement** |
|  |  |
| **Potential health benefits** |  |
| Detecting cancer at an early stage increases the likelihood of successful endoscopic treatment. | De detectie van kanker in een vroeg stadium verhoogt de kans op een succesvolle beperkte behandeling. |
| Early detection leads to a shift in the type of surgery (more EMRs/ESDs, fewer esophagectomies) and therefore fewer (major) complications. | Vroege opsporing leidt tot verschuiving van soort operaties (meer EMRs/ESDs, minder oesofagus-cardia resecties) en daardoor minder (grote) complicaties. |
| Early detection of (precancerous) esophageal carcinoma will lead to fewer deaths. | Het vroeger opsporen van (voorstadia van) slokdarm carcinoom zal leiden tot minder overlijdens. |
| Preventing cancer in the screening population may improve the quality of life of individuals. | Het voorkomen van kanker in de screeningspopulatie verbetert de kwaliteit van leven van individuen. |
| The introduction of a screening programme will reduce the incidence of synchronous esophageal cancer metastasis. | De invoering van een BVO [bevolkingsonderzoek] zal het aantal mensen met een synchroon gemetastaseerd EC verlagen. |
| May significantly reduce the incidence of esophageal adenocarcinoma. | Kan in belangrijke mate de incidentie van het adenocarcinoom van de slokdarm verminderen. |
| Increased opportunity for curative treatment, i.e., resection | Meer patiënten curatief te behandelen, resectie |
| Preventive treatments for Barrett esophagus would be an advantage. | Preventieve behandelingen bij Barrett zijn een voordeel. |
| Screening may facilitate early detection of the disease. | Screenen maakt het mogelijk ziekte vroegtijdig op te sporen. |
| The introduction of a screening programme will increase the quality of local excisions. | De invoering van een BVO [bevolkingsonderzoek] zal de kwaliteit van lokale excisies verhogen. |
|  |  |
| **Cost-effectiveness** |  |
| Screening for Barrett's esophagus with a non-invasive test aimed at detecting Barrett's esophagus with (high-grade) dysplasia may be cost-effective. | Screening op Barrett slokdarm met een niet-invasieve test gericht op het vinden van Barrett slokdarm met (hooggradige) dysplasie kan kosteneffectief zijn. |
| A screening programme that specifically aims to detect precursors (Barrett esophagus) is not cost-effective. | Een BVO dat zich specifiek richt op voorlopers (Barrett) is niet kosteneffectief. |
| The financial investment in screening will be recovered by preventing higher healthcare costs later on. | De gedane financiële investering in screening verdient zich terug door het voorkomen van latere hogere zorgkosten. |
| In my opinion, it is only cost-effective if there is a good pre-screening (analogous to, for example, the FiOBT). | Mijns inziens is het alleen kosteneffectief als er een goede prescreening is (analoog aan bv de FiOBT). |
| Not cost-effective in case of population screening. | Niet kosten effectief bij een landelijk screenings programma. |
| The cost-effectiveness of screening is related to the incidence of disease and the cost of the screening instrument. | De kosteneffectiviteit van screenen hangt samen met incidentie van ziekte en de kosten van het screeningsinstrument. |
| The price of screening is high due to material costs and the deployment of manpower. | De prijs van screening is door materiaalkosten en inzet van menskracht hoog. |
| The cost of screening may be reduced by alternative/new techniques and alternative form of organization. | De screeningskosten kunnen lager worden door andere / nieuwere technieken en andere organisatievorm. |
| It is unclear whether screening for BE/EAC is cost-effective. | Het is onduidelijk of deze screening kosteneffectief is. |
| Costs are an important argument in introducing screening on a large scale. | Kosten zijn een belangrijk argument voor het wel dan wel niet invoeren van screening op grote schaal. |
| Expensive maintenance treatments for stage 1 esophageal cancer may limit cost-effectiveness of screening. | Screening op slokdarmkanker zal kosteneffectief zijn als de patiënten met een stadium 1 slokdarmkanker geen dure onderhoudsbehandelingen krijgen. |
| Reimbursement of screening and subsequent treatment by the same authority will increase support for screening. | Het dragen van de kosten van de screening en die van de behandeling van ontstane ziekte door dezelfde instantie, zal het draagvlak voor deze screening vergroten. |
| Expressing benefit and risk in a cost-effectiveness ratio may be interesting from an accounting point of view, but hardly from a public health point of view. | Nut en risico uitdrukken in een kosteneffectiviteitsratio is vooral boekhoudkundig interessant, gezondheid kundig niet of nauwelijks. |
|  |  |
| **Design screening method** |  |
| The screening method should be highly specific to avoid having too many people undergoing endoscopy. | Methode screening moet ook hoge specificiteit hebben om te voorkomen dat teveel mensen endoscopie moeten ondergaan. |
| The screening method should be highly sensitive to avoid missing too many people. | Methode van screening moet hoge sensitiviteit hebben om te voorkomen dat teveel mensen worden gemist. |
| Besides test characteristics such as specificity and sensitivity, the willingness of the public to undergo this test largely determines the success of a screening programme. | Niet alleen test karakteristieken zoals specificiteit en sensitiviteit maar juist ook de bereidwilligheid van cliënten om die test te ondergaan bepaalt het succes van het BVO [bevolkingsonderzoek]. |
| The actual ability of less invasive screening methods compared to oesophago-gastro-duodenoscopy to distinguish ill and non-ill persons is essential. | Het daadwerkelijke vermogen van minder invasieve screeningsmethoden ten opzichte van een oesophago-gastro-duodenoscopie om zieke en niet-zieke personen te onderscheiden is essentieel. |
| Patients have a preference for non-invasive screening tests. | Patiënten hebben voorkeur voor non invasief onderzoek. |
| Until there is a good non-invasive alternative to endoscopy, screening for esophageal cancer is not attractive. | Zolang er geen goed niet-invasief alternatief voor endoscopie is, is screening op slokdarmkanker niet aantrekkelijk. |
| We don't have enough experience with new screening methods for large-scale screening. | Er is nog niet genoeg ervaring met nieuwe screeningsmethoden voor brede screening. |
| Esophagogastroduodenoscopy is not suitable for large-scale screening. | Oesophago-gastro-duodenoscopie is niet geschikt voor screening op grote schaal. |
| Individuals will be reluctant to participate because they find the test scary or burdensome. | Mensen zullen terughoudend zijn om mee te doen omdat ze het onderzoek eng of belastend vinden. |
| Screening should be as non-invasive as possible, transnasal endoscopy is too invasive for individuals without symptoms in my opinion. | In ieder geval zoveel mogelijk non-invasief, nasoscopie is denk ik te invasief voor screenees zonder klachten. |
| It is unclear whether individuals should be screened repeatedly. | Het is onduidelijk of personen herhaaldelijk gescreend moeten worden. |
| An exit strategy from the screening in case of certain findings is crucial. | Een exit-strategie uit de screening bij bepaalde bevindingen is cruciaal. |
| Applying screening techniques in primary care saves a large number of endoscopies. | Toepassen van screening technieken in de eerste lijn scheelt groot aantal gastroscopieën. |
| Standard endoscopy is human-unfriendly. | De standaard flexibele endoscopie is mens-onvriendelijk. |
| Esophageal cancer is too little a problem among the public to generate enough enthusiasm to participate in screening. | Slokdarmkanker leeft te weinig als probleem onder de bevolking om mensen genoeg te enthousiasmeren aan screening deel te nemen. |
| A non-invasive test is harmful rather than useful because it lowers the threshold to start screening (even at low risk of cancer). | Een niet-invasieve test is eerder schadelijk dan nuttig, omdat het de drempel verlaagt om (ook al) bij lager risico te screenen. |
| Patients with severe disease are unable to refuse esophageal cancer screening. | Patiënten met een ernstige ziekte zijn niet in staat om slokdarmkankerscreening te weigeren. |
| A non-invasive test, such as a breath test, reinforces the illusion of low-risk screening and is therefore harmful rather than helpful. | Een niet-invasieve test, zoals een ademtest, versterkt de illusie van laag-risico screening en is daarom eerder schadelijk dan nuttig. |
|  |  |
| **Legitimacy** |  |
| Without mortality reduction (or other clinically relevant outcomes), detection of precursors (Barrett) and disease (esophageal cancer) only results in risk and even harm, but not in benefit! | Zonder sterftereductie (of objectief andere klinisch relevantie uitkomsten) is detectie van voorstadia (Barrett) en ziekte (slokdarmkanker) alleen maar risico en zelfs schade, in ieder geval geen nut! |
| Most patients with Barrett esophagus do not develop esophageal cancer or dysplasia. | De meeste patiënten met Barrett ontwikkelen geen slokdarm kanker of dysplasie. |
| Invasive screening for esophageal cancer among high-risk individuals should only be done if these people are well aware of the risks of both screening and refraining from screening. | Invasief screeningsonderzoek van slokdarmkanker onder individuen met een hoog risico kan alleen als deze mensen goed op de hoogte zijn van de risico’s van wel van niet screenen. |
| As experience with this is limited, we do not know whether the advantages of screening outweigh the disadvantages. | Omdat hier nog weinig ervaring mee is, weten we nog niet goed of de voordelen van screening opwegen tegen de nadelen. |
| Decrease in esophageal cancer mortality will be limited despite screening because asymptomatic patients with an increased risk of esophageal cancer are still missed. | Ondanks screening slechts geringe daling op mortaliteit van slokdarmkanker doordat asymptomatische patiënten met verhoogd risico op slokdarmkanker nog steeds gemist worden. |
| Decrease in esophageal cancer incidence will be limited despite screening because asymptomatic patients with an increased risk of esophageal cancer are still missed. | Ondanks screening slechts geringe daling op incidentie van slokdarmkanker doordat asymptomatische patiënten met verhoogd risico op slokdarmkanker nog steeds gemist worden. |
| Screening can be helpful to filter patients at risk from the large group with reflux complaints. | Screening kan behulpzaam zijn om patiënten at risk te filteren uit de grote groep met refluxklachten. |
| It is unclear whether the disease is sufficiently recognizable at an early stage. | Het is onduidelijk of de ziekte voldoende herkenbaar is in een vroeg stadium. |
| The low incidence and low mean survival are barriers in screening for esophageal cancer | De lage incidentie samen met de lage gemiddelde overleving spreekt tegen screenen op slokdarmkanker |
| It is difficult to estimate the feasibility due to the knowledge gap of BE progression at a population level (interval cancers). | Gebrek aan inzicht over progressie van BE [Barrett slokdarm] op populatieniveau maakt haalbaarheid moeilijk in te schatten (intervalkankers). |
| Insight into the incidence of BE in the screening population is insufficient, therefore pilot screening studies are needed. | Er is onvoldoende inzicht in de incidentie van BE [Barrett slokdarm] in de screeningspopulatie, hiervoor zijn pilot studies nodig. |
| The risk of Barrett's esophagus and esophageal cancer is accompanied by other comorbidities, which may limit the health benefits of screening. | De kans op Barrett slokdarm en slokdarmkanker gaat gepaard met andere comorbiditeiten wat de gezondheidswinst als gevolg van screening zeer kan beperken. |
| Early cancer detection may result in a longer lifetime knowing you have cancer, but not a longer lifetime. | Eerdere ontdekking van kanker kan betekenen langer leven met de kennis dat je kanker hebt, maar niet langer leven. |
| The prevalence of esophageal cancer is too low to initiate population screening. | Slokdarmkanker is te weinig prevalent om populatiescreening te starten. |
| Randomized research into mortality reduction effectiveness must be conducted before screening can be implemented. | Voordat slokdarmkanker screening geïmplementeerd kan worden moet eerst gerandomiseerd onderzoek gedaan worden naar de effectiviteit ervan in het terugdringen van sterfte aan slokdarmkanker. |
| Great benefit to few people. | Screening op slokdarmkanker zal voor weinig mensen veel winst opleveren. |
| Screening uptake is related to disease incidence, disease severity and test burden | Opkomst van screenen hangt samen met incidentie van de ziekte, de ernst van de ziekte en de belasting van de screening |
| The dismal prognosis of this cancer justifies early detection. | De sombere prognose van deze kanker rechtvaardigt vroege opsporing. |
| Preventing cancer in the screening population improves public health. | Het voorkomen van kanker in de screeningspopulatie verbetert de volksgezondheid. |
| As the incidence of esophageal cancer is increasing rapidly in the Netherlands, the potential for screening has grown. | Slokdarmkanker is een sterk groeiende ziekte in Nederland waardoor de potentie voor screening sterk is toegenomen. |
| Even if it is detected through screening, people usually still die of esophageal cancer. | Ook als het met screening wordt ontdekt, sterft men desondanks meestal toch aan slokdarmkanker |
| Screening always leads to risk and harm, sometimes to some good and rarely to more good than risk and harm. | Alle screening leidt tot risico en schade, soms ook tot wat goeds en zelden tot meer goed dan risico en schade. |
| It might be useful only for people who would die of esophageal cancer without population screening. | Alleen voor mensen die zonder bevolkingsonderzoek zouden sterven aan slokdarmkanker kan het nuttig zijn, met nadruk alléén voor hen en met nadruk en kán. |
| The more complex the benefit-risk ratio is, the higher the threshold for screening should be. | Hoe complexer de nut-risicoverhouding is, hoe hoger de drempel moet zijn en dat spreekt tegen een laagdrempeligere screening. |
| Adding an easily accessible screening test to an unfavorable benefit-risk ratio is like a fish trap with bait, or (for music lovers) like Hotel California: you can enter, but never leave. | Aan een niet gunstige nut-risicoverhouding een laagdrempelige screeningstest toevoegen, is als een vis fuik met aas, of beter nog (voor de muziekliefhebbers) als Hotel California: you can enter, but never leave. |
| Other findings or missing them can be a reason to refrain from screening for esophageal cancer, but should never be a reason to screen for 'just anything'! | Andere bevindingen of het missen daarvan kunnen wel reden zijn om af te zien van screenen (op slokdarmkanker), maar kunnen nooit reden zijn om ‘daar dan ook maar’ op te screenen! |
| The complexity of the benefit-risk ratio should be directly proportional to the complexity of the screening. | De complexiteit van de verhouding tussen nut en risico moet recht evenredig zijn met de complexiteit van de screening. |
| Benefit and risk are incomparable values | Nut en risico zijn onvergelijkbare grootheden |
| A relatively simple benefit-risk ratio does not justify low-threshold screening, because the screening is part of the benefit-risk ratio – a snake biting its tail. | Een relatief eenvoudige nut-risicoverhouding rechtvaardigt niet een laagdrempelige screening, omdat de screening onderdeel is van de nut-risicoverhouding – een slang die in zijn eigen staart bijt. |
|  |  |
| **Capacity** |  |
| Adequate education of general practitioners and patients is essential. | Adequate voorlichting aan huisartsen en patiënten ten aanzien van screening op Barrett oesophagus/ vroeg carcinoom is essentieel. |
| Less-invasive screening methods (eNose, esosponge) may be suitable for large-scale screening. | Minder invasieve screeningsmethoden (eNose, esosponge) zouden geschikt kunnen zijn voor screening op grote schaal. |
| Undergoing a screening test in primary care is more accessible for patients than endoscopy in the hospital. | Screening op Barrett oesofagus/vroeg carcinoom bij huisarts is laagdrempeliger voor patiënten dan gastroscopie in ziekenhuis. |
| The current endoscopy capacity is insufficient for the expected increase in Barrett esophagus surveillance. | De huidige endoscopie capaciteit is onvoldoende voor de te verwachten toename in surveillance. |
| A limiting factor may be the increase in work for gastroenterologists and pathologists. | Belemmerende factor kan zijn de toename van werk voor MDL-arts en patholoog. |
| A home testing kit (and not in the hospital or primary care) is preferable because of the burden on the health care system. | Een screeningsmethode voor thuis (en niet in ziekenhuis of bij huisarts) verdient de voorkeur i.v.m. belasting van zorgsysteem. |
| The national capacity for endoscopic treatment of early carcinomas is not sufficient to introduce a screening programme. | De landelijke capaciteit voor endoscopische behandeling vroeg carcinomen biedt geen ruimte voor een screeningsprogramma. |
| Shouldn't we focus on prevention (smoking/alcohol)? | Zou inzetten op preventie (roken/alcohol) niet hoger op de agenda moeten staan? |
| Setting up a national system (such as the bowel cancer screening program, but a more specific group for this) ensures that screening for Barrett's esophagus/early carcinoma at the GP can be successful. | Het opzetten van een landelijk systeem (bijvoorbeeld zoals screeningorganisatie BVO darmkanker, echter hiervoor een specifiekere groep) zorgt dat screening op Barrett oesophagus/ vroeg carcinoom bij huisarts succesvol kan zijn. |
| How are we going to handle the increase endoscopies? Do we have sufficient capacity in gastroenterologists or do we need to train more? We should avoid long waiting lists! | Hoe gaan we de toeloop voor endoscopieën aanvliegen? Voldoende capaciteit MDL nu al of moeten er meer worden opgeleid? Ook voorkomen lange wachtlijsten! |
| Using artificial intelligence for pill/sponge screening instrument histology assessment. | Artificial intelligence inzetten voor de beoordeling van de histologie afkomstig van een pil/ spons screening instrument. |
| Screening at the GP will burden the healthcare system too much. | Screening bij de huisarts zal het zorgsysteem teveel belasten/ is teveel werk voor huisartsen. |
| Applicable within 1.5 line care and the deployment of nursing specialists or physician assistants. | Mooi toepasbaar binnen 1,5-lijns zorg met een VS [verpleegkundig specialist] of PA [physician assistent] die deze patiëntengroep ziet. |
| Esophageal cancer screening should be performed in primary care. | Slokdarmkankerscreening moet worden uitgevoerd in de eerste lijn. |
| Treating a Barret's esophagus is well done in general practice. | Behandelen van een Barrett oesophagus wordt goed gedaan in de huisartsenpraktijk. |
| Ownership of screening within primary care will result in referral delay. | Als de vroege screening bij de huisarts wordt ondergebracht, kom je te laat bij de specialist. |
|  |  |
| **Harms of screening** |  |
| Detection of Barrett's esophagus without dysplasia leads to overdiagnosis of patients who would never have developed esophageal cancer in their lives. | Detectie van Barrett slokdarm zonder dysplasie leidt tot veel overdiagnose van patiënten die nooit in hun leven slokdarmkanker zouden hebben ontwikkeld. |
| The risk of overtreatment which results in more complications. | Er bestaat een risico op overbehandeling met als gevolg toename complicaties. |
| Detection of Barrett's esophagus without dysplasia results in a lifetime of largely unnecessary surveillance endoscopies. | Detectie van Barrett slokdarm zonder dysplasie heeft een leven lang van grotendeels onnodige endoscopische controles tot gevolg. |
| Screening for esophageal cancer by detecting Barrett's esophagus often leads to false reassurance. | Screenen op slokdarmkanker middels detectie van een Barrett-slokdarm leidt vaak tot valse geruststelling. |
| Disadvantages: complications of biopsies. | Nadelen: complicaties van biopten. |
| A barrier to detecting more early stages is that imaging and endoscopic techniques are not yet able to properly distinguish between T1 and T2 carcinomas, thus limiting good treatment advice (surgery vs endoscopic resection, pre-treatment in case of surgery). | Belemmerende factor van meer vroege stadia is dat de beeldvormende en endoscopische technieken nog niet goed onderscheid kunnen maken tussen T1 en T2, dit belemmert in de beste keuze voor de patiënt (chirurgie versus endoscopische resectie versus wel/ geen voorbehandeling bij eventuele chirurgie). |
| An esophagectomy is quite disabling. | Een buismaag is behoorlijk invaliderend. |
| The introduction of a screening programme will increase the quality of pathological assessment. | De invoering van een BVO [bevolkingsonderzoek] zal de kwaliteit van de pathologische beoordeling verhogen. |
| Screening for Barrett's esophagus will result in anxiety among reflux patients. | Screening op Barrett oesophagus zal voor veel onrust zorgen onder reflux patiënten. |
| Patients with Barrett's esophagus use proton pump inhibitors for a lifetime. Side effects? Cost? Medicalization? | Barrett betekent levenslang PPI [maagzuurremmer]. Bijwerkingen? Kosten? Medicalisatie? |
| Screening for Barrett's esophagus will be reassuring for reflux patients. | Screening op Barrett oesophagus zal geruststellend zijn voor reflux patiënten. |
| The introduction of a screening programme will increase the quality of endoscopic assessment. | De invoering van een BVO [bevolkingsonderzoek] zal de kwaliteit van de endoscopie verhogen. |
|  |  |
| **General considerations** |  |
| Should also go hand in hand with more effective surveillance in patients with known Barrett (less, better, selection of a population at risk, reduction of costs). | Moet ook hand in hand gaan met doelmatiger surveillance bij patiënten met bekende Barrett (minder, beter, selectie risicopopulatie, reductie van kosten). |
| Don't start considering screening for esophageal cancer until Wilson & Junger’s 10 criteria are genuinely fulfilled, it's not a tick list. | Begin pas screening op slokdarmkanker te overwegen als alle seinen voor de 10 criteria van Wilson & Jungner volledig op groen staan, het is geen afvinklijstje. |
| Screening for Barrett's esophagus/early carcinoma in primary care will save cost compared to endoscopy in the hospital. | Screening op Barrett oesophagus/vroeg carcinoom bij huisarts is kosten besparend ten opzichte van gastroscopie in ziekenhuis. |
| Most esophageal carcinomas are discovered in patients not known to have Barrett's esophagus… | De meeste slokdarmcarcinomen worden ontdekt bij patiënten die niet bekend zijn met Barrett… |
| Given the freedom for individuals to choose a health insurer, including switching to another insurer, the screening will need to be publicly funded. | Gezien de verzekeringsstructuur met de vrijheid van keuze van zorgkostenverzekeraar inclusief overstappen van de client naar een andere verzekeraar, zal de screening bekostigd moeten worden uit het Rijksprogramma voor bevolkingsonderzoeken. |
| Squamous cell carcinoma should also be included in a screening program. | Plaveiselcelcarcinoom dient ook meegenomen te worden in een screeningsprogramma. |
| Screening for esophageal cancer by detecting Barret's esophagus is just health care on indication. | Screenen op slokdarmkanker middels detectie van een Barrett-slokdarm is gewoon zorg op indicatie. |
| Patients with Barrett Esophagus can come to the GP when they have symptoms, so why should we screen? | Patiënten met Barrett komen toch naar de huisarts i.v.m. klachten, dus waarom screenen? |
|  |  |
| **Target population** |  |
| It is unclear whether a relevant risk group can be defined for this disease. | Het is onduidelijk of een juiste risicogroep te definiëren is voor deze ziekte. |
| A good population-based study should be carried out to determine which risk groups are relevant. | Er dient een goede population based studie plaats te vinden welke risicogroepen relevant zijn. |
| A clear guideline should be written about who is eligible for esophageal cancer screening. | Er moet een duidelijke richtlijn worden geschreven over wie in aanmerking komt voor slokdarmkankerscreening. |
| Defining the age range for screening is essential. | Afbakening van de leeftijdscategorie voor screening is essentieel. |
| Population groups at risk (alcoholism) are probably difficult to reach. | Echte risicogroepen (alcoholisme) zijn waarschijnlijk moeilijk bereikbaar. |
| Screening based on risk factors (age > 50, overweight, white race, etc?) | Screening op geleide van risicofactoren (leeftijd > 50, overgewicht, blanke ras, etc. ?) |
| Patients with GERD are open to esophageal cancer screening. | Patiënten met GERD [gastro-oesofageale refluxziekte] hebben behoefte aan/staan open voor slokdarmkankerscreening. |
| Should be offered to people with chronic reflux symptoms. | Moet worden uitgevoerd bij mensen met chronische refluxklachten. |
| Selecting the population at risk is complicated and requires training for doctors or other healthcare personnel. | Het selecteren van de risicogroep is ingewikkeld en vergt training van artsen of ander zorgpersoneel. |
| For all patients with more than 10 years of PPI use and no previous endoscopy. | Alle patiënten met langer dan 10 jaar PPI [maagzuurremmer] gebruik en geen eerdere endoscopie. |
| The inclusion criterion ‘white male’ is a form of ethnic discrimination, which is politically questionable. | Het inclusiecriterium: witte man, is een vorm van etnische discriminatie, welke politiek gezien twijfelachtig is. |
| The narrow definition of the screening population may result in non-eligible individuals demanding a screening test (like breast cancer screening at an older age). | De te krappe definitie van de screeningspopulatie heeft als risico dat meer mensen deelname gaan eisen (vergelijk borstkankerscreening op oudere leeftijd). |
| The GP should select patients eligible for screening. | De huisarts moet selecteren welke patiënten in aanmerking komen voor slokdarmkankerscreening. |
| Obesity is a risk factor, and waist-hip ratio is more relevant than BMI. | Overgewicht is een risicofactor, waarbij taille-heup ratio de voorkeur verdient boven de BMI. |
| Not appropriate in case of dysphagia, this warrants direct referral for endoscopy. | Niet toepasbaar bij dysfagie klachten, dan gastroscopie als eerste keuze. |
| Risk population: using data on alcohol and nicotine abuse in hospital information systems. | Risico populatie: koppelen aan gegevens over alcohol- en nicotine abusus in ZIS [ziekenhuis informatie systeem]. |
| Combining BE/EAC screening with other screening programs (breast of colorectal cancer), possibly only for individuals with a high BMI or alcohol/nicotine abuse. | Koppelen van een test aan andere BVO (mammacarcinoom en CRC), eventueel alleen die mensen met hoog BMI en eventueel gecombineerd met intoxicaties. |
